# Maternal folate deficiency causes inhibition of mTOR signaling, down-regulation of placental amino acid transporters and fetal growth restriction in mice

**DOI:** 10.1038/s41598-017-03888-2

**Published:** 2017-06-21

**Authors:** Fredrick J. Rosario, Peter W. Nathanielsz, Theresa L. Powell, Thomas Jansson

**Affiliations:** 1 Division of Reproductive Sciences, Department of Obstetrics and Gynecology, University of Colorado Anschutz Medical Campus, Aurora, CO 80045 USA; 2 Department of Animal Science, University of Wyoming, Laramsie, WY 82071 USA; 3 Southwest National Primate Research Center, San Antonio, TX 78249 USA; 4 Section of Neonatology, Department of Pediatrics, University of Colorado Anschutz Medical Campus, Aurora, CO 80045 USA

## Abstract

Maternal folate deficiency is linked to restricted fetal growth, however the underlying mechanisms remain to be established. Here we tested the hypothesis that mTOR functions as a folate sensor *in vivo* in mice and that maternal folate deficiency inhibits placental mTOR signaling and amino acid transporter activity and causes fetal growth restriction. Folate deficient mice had lower serum folate (−60%). In late pregnancy, fetal weight in the folate deficient group was decreased (−17%, p < 0.05), whereas placental weight, litter size and crown rump length were unaltered. Maternal folate deficiency inhibited placental mTORC1 and mTORC2 signaling and decreased trophoblast plasma membrane System A and L amino acid transporter activities and transporter isoform expression. Folate deficiency also caused a decrease in phosphorylation of specific functional readouts of mTORC1 and mTORC2 signaling in multiple maternal and fetal tissues. We have identified a novel specific molecular link between maternal folate availability and fetal growth, involving regulation of placental mTOR signaling by folate, resulting in changes in placental nutrient transport. mTOR folate sensing may have broad biological significance because of the critical role of folate in normal cell function and the wide range of disorders, including cancer, that have been linked to folate availability.

## Introduction

Folate is critical for normal fetal development and growth and maternal folate deficiency is associated with poor pregnancy outcomes^1, 2^. Periconceptional folate deficiency is associated with neural tube defects (NTDs)^3^ and animal experiments, epidemiological studies and interventional trials have demonstrated that folate supplementation decreases the incidence of these structural fetal malformations^4–6^. Low maternal folate levels are also linked to restricted fetal growth^2, 7–9^ and preterm birth^10^, and two cohort studies showed that supplementation with high-dose folic acid (3.7–5 mg/d) reduced the risk of low birth weight and preterm delivery^11, 12^.

It is now well-established that exposures during early life modulate the risk of developing non communicable diseases in childhood and in adult life, a concept known as the developmental origins of health and disease (DOHaD) or fetal programming^13, 14^. For example, there is substantial evidence for an association between low birth weight and risk to develop type-2 diabetes, coronary heart disease, and hypertension later in life, which has been attributed to poor nutrition *in utero* ^15, 16^. Reduced folate availability has been implicated in fetal programming^17^, which may in part be mediated by link between folate deficiency and fetal growth restriction^7, 8^ and in part due altered methylation of critical genes. Low birth weight is a predictor for metabolic disease in later life^18^. Recently, folic acid supplementation during pregnancy in a generally undernourished population was shown to reduce the prevalence of metabolic syndrome (MS) in children at 6 to 8 years of age^19^. Furthermore, folate supplementation in pregnant rats modified growth patterns and the metabolic response to fasting in adult offspring^20^, providing further evidence that maternal folate intake during pregnancy affects metabolic health in offspring.

Folate is a water-soluble B vitamin that is essential for the synthesis of purine and thymidine nucleotides, which are needed for DNA replication and repair^21^. In addition, folate is a critical methyl donor for DNA methylation, which is a key mechanism of epigenetic regulation. Although impaired DNA synthesis/repair and gene methylation are generally believed to explain the association between folate deficiency and poor pregnancy outcomes, the mechanistic links remain elusive.

The mechanistic target of rapamycin (mTOR) is master regulator of cell growth, aging, ribosome biogenesis, protein synthesis, actin-cytoskeletal organization, autophagy and metabolism^22^. mTOR forms two distinct heteromeric complexes, mTOR Complex 1 (mTORC1) and mTORC2. mTORC1 contains mTOR, raptor (regulatory associated protein of mTOR), mLST8 and PRAS40^23^, while mTOR in mTOR Complex 2 associates with rictor (rapamycin-insensitive companion of mTOR), mLST8, mSin1 and protor^24^. Thus, raptor and rictor constitute specific components of mTORC1 and mTORC2, respectively. We have previously demonstrated that both mTORC1 and mTORC2 are powerful positive regulators of placental amino acid transporters^25–29^. mTORC1 modulate System A and System L amino acid transport in primary human trophoblast cells at the posttranslational level by regulating Nedd4-2 mediated ubiquitination, which influences the trafficking of specific amino acid transporter isoforms to the plasma membrane^28^. Placental mTORC1 activity is inhibited in human IUGR^25, 29, 30^ and activated in placentas of large babies born to obese mothers^31^. Furthermore, placental mTORC1 activity has been reported to be decreased in hyperthermia-induced IUGR in the sheep^32^, in response to a maternal low protein diet in the rat^33^ and following maternal calorie restriction in the baboon^34^ and activated in a mouse model of maternal obesity associated with fetal overgrowth^35, 36^. Importantly, placental amino acid transport has been reported to be changed in the same direction as mTOR signaling in these clinical conditions and animal models of altered fetal growth^29, 31, 33–35, 37–39^. Collectively, this data suggest that trophoblast mTOR signaling regulates fetal growth by modulating placental nutrient transport. mTORC1 signaling is regulated by a multitude of upstream signals, including amino acids, ATP, glucose, oxygen and growth factors^40–42^. We recently reported that mTOR signaling functions as a folate sensor in primary human trophoblast cells, representing a previously unknown molecular mechanism by which folate regulates trophoblast cell function^43^. Folate sensing by mTOR in PHT cells involves both mTOR Complex 1 and 2 and requires the proton-coupled folate transporter (PCFT)^43^. Herein, we tested the hypothesis that mTOR functions as a folate sensor *in vivo* in mice and that maternal folate deficiency inhibits placental mTOR signaling and placental amino acid transporter activity and causes fetal growth restriction.

## Materials and Methods

### Experimental Design (Mice)

#### Animals

Experiments were carried out in ICR mice in accordance with the ‘Principles of Laboratory Animal Care’ (1996) and with the approval of the Institutional Animal Care and Use Committee at the University of Texas Health Science Center, San Antonio. Weanling female ICR mice (*n* = 20) were housed individually in polypropylene cages with wire mesh bottom and maintained at 22 °C ± 2 °C under standard lighting conditions (12-h light/dark cycle). They were divided into two groups (*n* = 10 each) and fed a control diet (TD110609, Harlan, WI) with 2 mg folate/kg diet for 6 weeks *ad libitum* or the same diet deficient in folate (TD 00434, Harlan, WI). Detailed information on the composition of the control and folate deficient diets is shown in Table 1. All the animals had free access to deionized water. Daily food intake and weekly body weights were determined. At the end of 6 weeks of feeding, blood was collected from the supraorbital sinus to determine serum folate concentrations and blood glucose. Subsequently female mice were mated with control males (2 females to 1 male) and the day a vaginal plug was detected was defined as embryonic day (E) 0.5. Animals were maintained on their respective diets throughout gestation. At E18.5, dams were euthanized for collection of blood and tissue samples.

**Table 1:** Dietary composition of control and folic acid deficient diet.

Formula	Control diet	Folic acid deficient diet
Sucrose	357.1526	357.1526
Corn Starch	150	150
Maltodextrin	160	160
Corn Oil	80	80
Cellulose	30	30
Mineral Mix, AIN-76	35	35
Calcium Phosphate, dibasic	3	3
^1^Protein:		
L-Alanine	3.5	3.5
L-Arginine HCL	12.1	12.1
L-Asparagine	6	6
L-Aspartic Acid	3.5	3.5
L-Cystine	3.5	3.5
L-Glutamic Acid	40	40
Glycine	23.3	23.3
L-Histidine HCL, monohydrate	4.5	4.5
L-Isoleucine	8.2	8.2
L-Leucine	11.1	11.1
L-Lysine HCL	18	18
L-Methionine	8.2	8.2
L-Phenylalanine	7.5	7.5
L-Proline	3.5	3.5
L-Serine	3.5	3.5
L-Threonine	8.2	8.2
L-Tryptophan	1.8	1.8
L-Tyrosine	5	5
L-Valine	8.2	8.2
Vitamin C, ascorbic acid coated (97.5%)	1.02	1.02
**Folic acid**	**0**.**002** (**2** **mg/kg**)	**0**.**000** (**0** **mg/kg**)
Biotin	0.0004	0.0004
Vitamin B_12_	0.03	0.03
Calcium Pantothenate	0.066	0.066
Choline Dihydrogen Citrate	3.5	3.5
Inositol	0.11	0.11
Vitamin K_3_, meandione	0.05	0.05
Niacin	0.099	0.099
Pyridoxine HCL	0.022	0.022
Riboflavin	0.022	0.022
Thiamin (81%)	0.022	0.022
Vitamin E, DL-alpha tocopheryl acetate (500 IU/g)	0.242	0.242
Vitamin A Palmitate (500,000 IU/g)	0.0396	0.0396
Vitamin D_3_, cholecalciferol (5000, 000 IU/g)	0.0044	0.0044
Ethoxyquin, antioxidant	0.02	0.02
^2^Protein (% by weight)	15.4	15.4
CHO (% by weight)	64.8	64.8
Fat (% by weight)	8	8
Kcal/g	3.9	3.9

^1^An amino acid defined diet used to eliminate background folic acid. This diet does not contain succinylsulfathiazole, which is used in quite a few folic acid deficient diets.

^2^Protein based on N X 6.25.

#### Biochemical measurements

Folate was measured in maternal serum by a microbiological assay using a commercially available kit (ALPCO Diagnostic Products, NH, USA), according to the manufacturer’s instructions. Blood glucose was measured in triplicate using a One Touch Ultra-2 (Life Scan Inc, Milpitas, CA, USA).

#### Tissue collection

Dams were fasted (4 hr) and then euthanized at E18.5 by carbon dioxide inhalation. After laparotomy, fetuses and placentas were collected and quickly dried on blotting paper, any remaining fetal membranes were removed. Fetal weight, crown–rump length (CRL), measured with digital calipers, and placental weight were recorded for each pup. Pups were rapidly submerged in ice and decapitated by cervical dislocation. Maternal and fetal liver, heart, kidneys, and spleen were dissected out, weighed and immediately snap frozen in liquid nitrogen, and stored at −80 °C until further analysis. All placentas in each litter were pooled and washed in phosphate buffer saline and transferred to buffer D [250 mM sucrose, 10 mM Hepes-Tris, and 1 mM EDTA (pH 7.4) at 4 °C], protease and phosphatase inhibitor cocktail (Sigma-Aldrich Corp., St. Louis, MO, USA) was added at a dilution of 1:1000, and the mixture was homogenized using a Polytron (Kinematica, Bohemia, NY, USA).

#### Isolation of trophoblast plasma membranes (TPM)

TPM were isolated from frozen placental homogenates using differential centrifugation and Mg^2+^ precipitation as described^35, 44^. This protocol results in the isolation of the maternal-facing plasma membrane of trophoblast layer II of mouse placenta, and accumulating evidence suggests that this membrane is functionally analogous to the syncytiotrophoblast microvillus plasma membrane of the human placenta^45^. Protein concentration was determined using the Lowry assay (Bio Rad, CA). Using electron microscopy, it has been previously shown that alkaline phosphatase is localized to the apical (maternal facing) plasma membrane of trophoblast layer II (TPM) in the mouse^45^. Thus, the ratio of the alkaline phosphatase activity in the TPM fraction over the activity in the homogenate ais used to determine the enrichment of TPM. The mean alkaline phosphatase enrichment for TPM vesicles isolated from placentas of FD animals was not significantly different from the enrichment in TPM vesicles obtained from control placentas (Supplementary Figure 1).

#### TPM amino acid transporter activity measurements

The activity of System A and L amino acid transporters in TPM was determined using radiolabelled amino acids and rapid filtration techniques slightly modified from procedures previously described for human syncytiotrophoblast plasma membranes and mouse TPM^44^. TPM vesicles were preloaded by incubation in 300 mM mannitol and 10 mM Hepes-Tris, pH 7.4 overnight at 4 °C. Subsequently, TPM vesicles were pelleted and resuspended in a small volume of the same buffer (final protein concentration 5–10 mg ml^−1^). Membrane vesicles were kept on ice until immediately prior to transport measurements when samples were warmed to 37 °C. At time zero 30 μl vesicles were rapidly mixed (1:2) with the appropriate incubation buffer containing [^14^C] methyl-aminoisobutyric acid (MeAIB, 150 μM) with or without Na^+^ or [3 H] L-leucine (0.375 μM). Based on initial time course studies (0–30 s), uptake at 15 s was used in all subsequent experiments. Uptake of radiolabelled substrate was terminated by addition of 2 ml of ice cold PBS. Subsequently, vesicles were rapidly separated from the substrate medium by filtration on mixed ester filters (0.45 μm pore size, Millipore Corporation, Bedford, MA, USA) and washed with 3 × 2 ml of PBS. In all uptake experiments, each condition was studied in duplicate for each membrane vesicle preparation. Filters were dissolved in 2 ml liquid scintillation fluid (Filter Count, PerkinElmer, Waltham, MA, USA) and counted. Appropriate blanks were subtracted from counts and uptakes expressed as pmol x (mg protein)^−1^. Na^+^-dependent uptake of MeAIB (corresponding to system A activity) was calculated by subtracting Na^+^-independent from total uptakes. For leucine, mediated uptake was calculated by subtracting non-mediated transport, as determined in the presence of 20 mM unlabelled leucine, from total uptake.

### Western blot analysis

#### Placental homogenates

Protein expression and phosphorylation of well-established functional readouts in the mTORC1 and mTORC2 signaling pathways was determined in placental homogenates using commercial antibodies (Cell Signaling Technology, Boston, MA). In addition, we determined TPM protein expression of the System A amino acid transporter isoforms (SNAT) 1, 2 and 4, the System L amino acid transporter isoforms LAT1 and LAT2. A polyclonal SNAT2 antibody generated in rabbits was received as a generous gift from Dr V. Ganapathy and Dr P. Prasad at the University of Georgia, Augusta. Western blotting was carried out as previously described^33^. Analysis of the blots was performed by densitometry.

#### Maternal and fetal tissues

Snap frozen maternal and fetal organs were homogenized in ice-cold radioimuno precipitation assay buffer (0.5 mol/L Tris-Cl, pH 7.4, 1.5 mol/L NaCl, 10 mmol/L ethylenediaminetetraacetic acid [EDTA], 2.5% deoxycholic acid, 10% NP-40, protease inhibitor, and phosphatase inhibitor), by rapid agitation in the presence of beads and spun at 13 000 g at 4 °C for 10 minutes. mTORC1 and mTORC2 signaling activity in maternal and fetal tissue homogenates was assessed by determining total protein expression and phosphorylation of key downstream targets using Western blot and commercial antibodies (Cell Signaling Technology, Boston, MA).

#### Proximity ligation assay (PLA) and confocal microscopy

Placentas were serially cryosectioned at 3 μm and sections were stored at −80 °C, until ready for use. The sections were blocked using 5% new born calf (NCS) serum in PBS for 1 hour followed by incubation in anti-mTOR (anti-rabbit) and anti-LAMP-2 (anti-mouse) antibodies for 2 hours. PLA probes anti-rabbit PLUS and anti-mouse MINUS were diluted in Duolink dilution buffer and incubated in a pre-heated humidity chamber for 1 hour. This was followed by ligation, amplification and detection according to the Duolink *In Situ* Orange kit (Sigma-Aldrich) manufacturer’s protocol. Confocal microscopy was performed using Zeiss LSM 780 microscope at 63x magnification using oil immersion. Images were captured in the same laser settings with four Z-step of 0.4 um. In each section, at least ten randomly selected microscopic fields were used to calculate the number of mTOR-LAMP2 interaction positive sites (yellow dots) per mm^2^ and data were averaged to represent a single placenta.

### Experimental design (baboons)

#### Animals and diets

All procedures were approved by the Texas Biomedical Research Institute Institutional Animal Care and Use Committee and conducted in facilities approved by the Association for Assessment and Accreditation of Laboratory Animal Care. Baboons (Papio species) were housed in outdoor metal and concrete gang cages, each containing 10–16 females and 1 male. Details of housing, environmental enrichment and system for controlling and recording individual feeding have been described elsewhere^46^. Animals were fed Purina Monkey Diet 5038 (Supplementary Table 1, Purina, St. Louis, MO, USA). Females were mated, pregnancy confirmed by ultrasound at gestational day (GD) 30, subsequently animals subjected to maternal nutrient restriction (MNR) were fed 70% of the total food intake of contemporaneous controls on a per-kilogram basis as previously described in detail^47^.

#### Collection of tissue and blood samples

Cesarean sections were performed under isoflurane anesthesia at GD 165 (term 184). Briefly, animals were tranquilized with ketamine hydrochloride (10 mg/kg), intubated, and anesthetized using isoflurane (starting rate 2% with oxygen: 2.0 L/min). Conventional cesarean sections using standard sterile technique were performed as described previously^46^. At cesarean section, fetuses and placentas were towel dried and weighed. Postoperative analgesia was provided using buprenorphine (0.015 mg/kg/d as 2 doses) for 3 d^46^.

#### Maternal serum folate analysis

Serum was prepared and frozen at −80 °C until analysis of folate concentrations using an Immulite 1000 (Siemens Healthcare Diagnostics, Deerfield IL), according to the manufacturer’s instructions. Intra- and inter-assay coefficients of variation were 6.7 and 7.9% at 700 pg/ml.

### Western blot analysis

#### Placental homogenates

Placental mTORC1 and mTORC2 signaling functional readouts were measured as described for mice placental homogenates and reported elsewhere^34^.

#### Placental microvillus membrane system A and L amino acid transport activity and expression

Syncytiotrophoblast plasma microvillous membrane vesicles (MVM) were isolated as described in detail previously^48^, with minor modifications^34^. MVM system A and L amino acid transporter activity and isoform expression were measured as described previously^33, 49^ and reported elsewhere^34^. In the current study we determined the correlation between placental mTORC1 and mTORC2 signaling/amino acid transporter activity or expression^34^ and maternal serum folate and fetal weight^34^.

### Experimental design (humans)

This study was approved by the Institutional Review Board at the University of Texas Health Science Center at San Antonio (approval no. HSC20070723H). Women with uncomplicated singleton term (37–40 weeks’ gestation) pregnancies were recruited with written consent before undergoing scheduled cesarean deliveries, which routinely were scheduled at 39 weeks gestation. Informed consent was obtained from all subjects. These women had had one or more previous cesarean delivery and were either not a candidate for, or declined, a trial of labor. Gestational age was estimated from the date of the last menstrual period and confirmed by ultrasound dating.

Maternal venous blood samples were obtained prior to c-section. Serum was prepared and frozen at −80 °C until analysis of folate concentrations using an Immulite 1000 (Siemens Healthcare Diagnostics, Deerfield IL), according to the manufacturer’s instructions. Intra- and inter-assay coefficients of variation were 6.7 and 7.9% at 700 pg/ml. The placenta was collected within 15 min of delivery, the decidua basalis and chorionic plate were removed and villous tissue was dissected and rinsed in cold physiological saline. The villous tissue was transferred to cold buffer D (250 mM sucrose, 10 mM HEPES, pH 7.4) containing 1:100 dilution of protease and phosphatase inhibitors (Sigma-Aldrich, St. Louis, MO) and homogenized on ice with a Polytron (Kinematica, Luzern, Switzerland). The placental homogenates were frozen in liquid nitrogen and stored at −80 °C until further processing. MVM system A and L amino acid transporter activity was measured as described previously^48^.

#### Data presentation and statistics

In mice, placental and fetal data were averaged within each litter and the litter mean was used as the observation for that dam. Thus, n represents the number of litters. Data are presented as means ± S.E.M or + S.E.M. Statistical significance of differences between control and experimental groups was assessed using Two-way repeated-measures (RM) ANOVA or Student’s t-test. A P value < 0.05 was considered significant.

## Results

### Dietary folate restriction before pregnancy results in decreased serum folate levels

**Figure 1:**
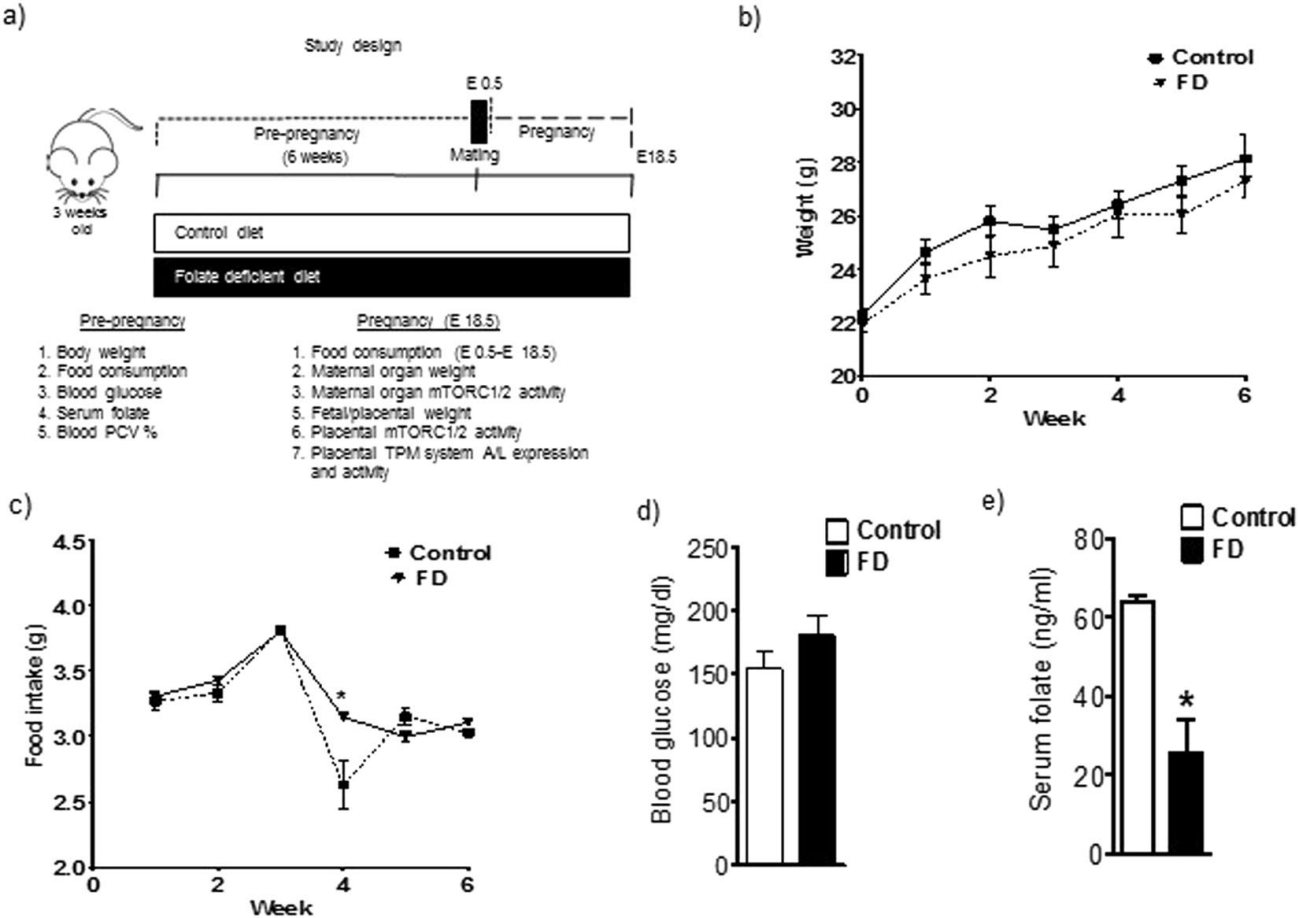
Effect of dietary folate restriction before pregnancy on body weight gain, food consumption and biochemical parameters in mice. (**a**) Study design illustrating the period of folate deficiency in mice. (**b**) ^#^Preconceptional body weight gain and (**c**) food intake were largely unaffected. (**d**) Fasting blood glucose was not influenced by folate deficiency but (**e**) serum folate levels were decreased by feeding a folate deficient diet for 6 weeks. Values are given as mean + SEM; *P < 0.05 vs. control; unpaired Student’s t-test; n = 6–10/each group; ^#^Two-way repeated-measure ANOVA and post-hoc pairwise comparisons with Bonferroni correction (diet × weight) did not reveal any significant interaction between folate diet and weight gain at different time points (F = 0.958, p = 0.45, n = 10 each group).

Dietary folate restriction between 3 and 9 weeks of age in female mice (Fig. 1a) did not influence body weight gain (Fig. 1b), food intake (Fig. 1c) or fasting blood glucose (Fig. 1d). The serum folate concentration was decreased by 60% after 6 weeks of dietary folate restriction (p = 0.0004, n = 8/each group, mean ± SEM, Fig. 1e).

### Effect of maternal folate deficiency on placental and fetal growth in mice

**Figure 2:**
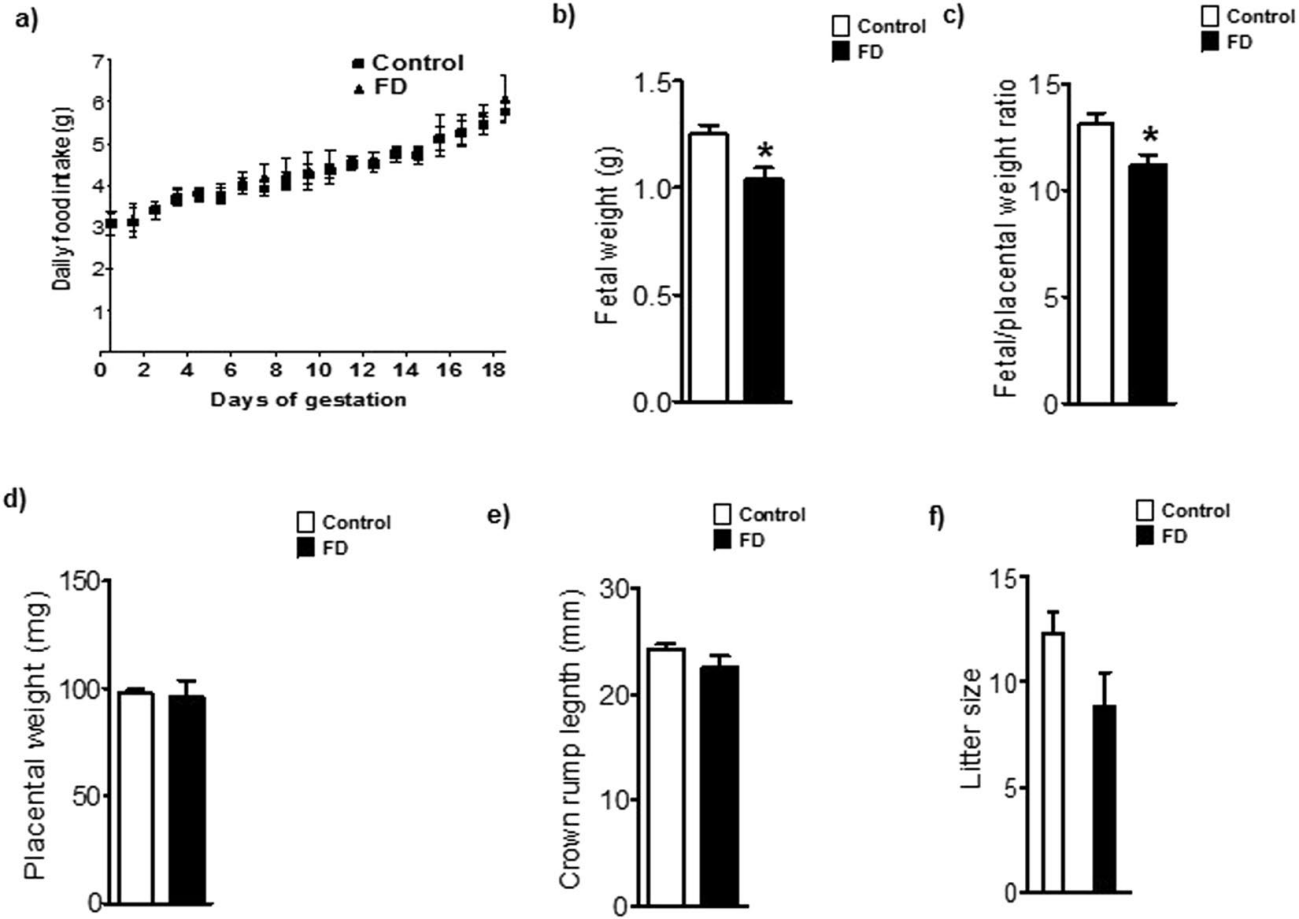
Effect of folate deficiency on food consumption, fetal and placental weights in mice. Feeding a folate deficient diet prior to and during pregnancy did not influence daily food intake (**a**) but did decreased fetal weight (**b**) and fetal/placental weight ratio (**c**). In addition, folate deficiency did not affect placental weight (**d**) crown rump length (**e**), or litter size (**f**). Placental and fetal data were averaged within each litter and the litter mean was used as the observation for that dam. Thus, n represents the number of litters. Values are given as mean + SEM; *P < 0.05 vs. control; unpaired Student’s t-test; n = 6–7.

Low maternal serum folate is associated with restricted fetal growth in pregnant women^1, 2, 50^, providing the rationale to examine the effects of maternal folate deficiency in mice on fetal-placental growth characteristics. Food intake during pregnancy was comparable between control and folate deficient groups (Fig. 2a). At E18.5, fetal weight (−17%, p < 0.05, Fig. 2b) and fetal/placental weight ratio (−14%, p < 0.05, Fig. 2c) were decreased in the folate deficient group, whereas placental weight, crown rump length and litter size were unaltered (Fig. 2d–f).

### Maternal folate deficiency inhibits placental mTORC1 signaling in mice

**Figure 3:**
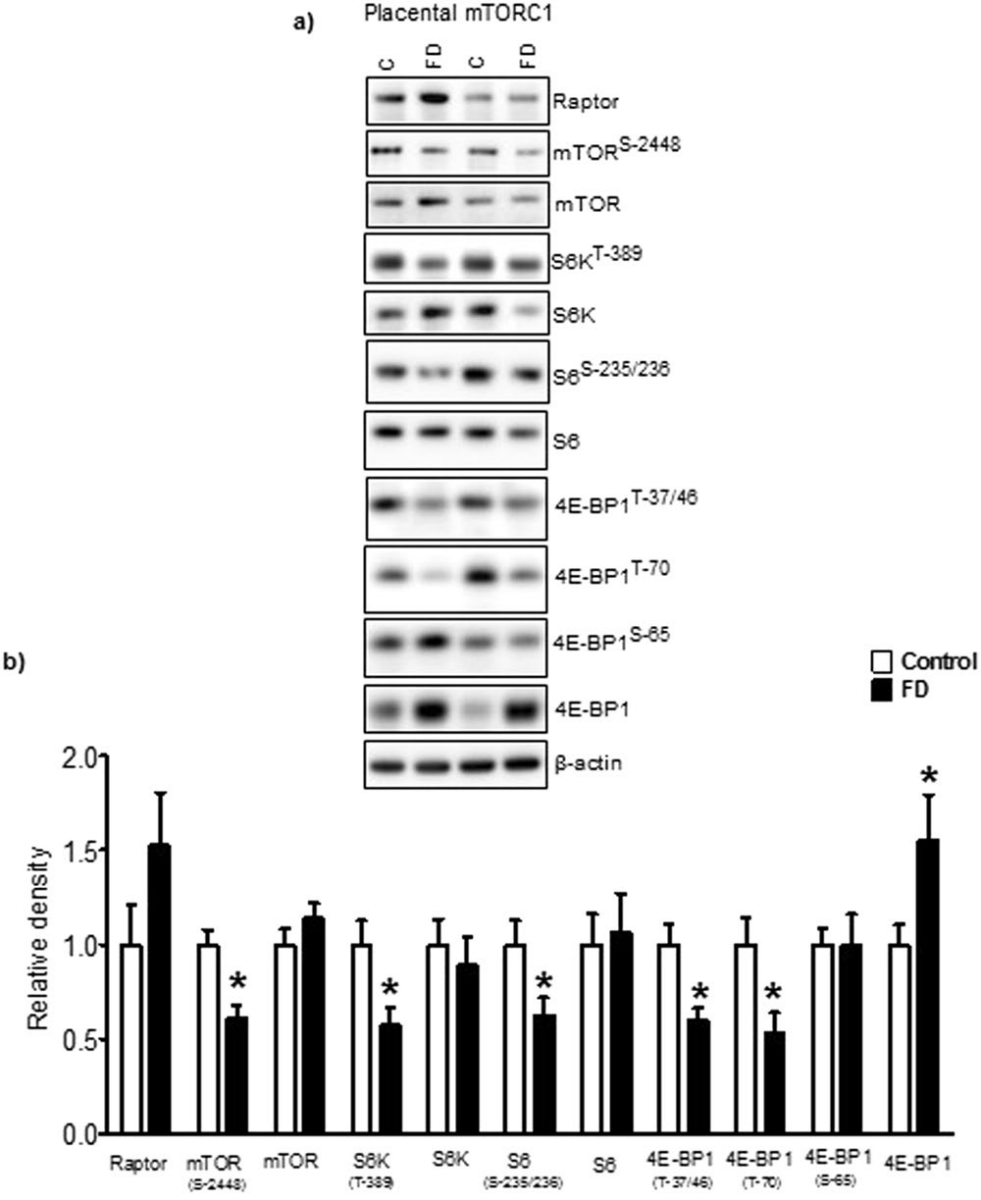
Maternal folate deficiency in mice inhibits placental mTORC1 signaling. (**a**) Placental mTORC1 signaling was inhibited in response to maternal folate deficiency. Histogram (**b**) summarizes the immunoblot data of placental mTORC1. Values are given as mean + SEM; *P < 0.05 vs. control; unpaired Student’s t-test; n = 6–7. Full-length gels and blots are included in the Supplementary Information File.

Raptor interacts with mTOR to form a nutrient-sensitive complex that signals to the cell growth machinery^51^. We studied the raptor protein expression in placental homogenates of the control and folate deficient mice. Raptor protein expression in the placenta was not significantly different in control and folate deficient mice (Fig. 3a,b). Upon activation, mTOR is phosphorylated on several residues, including T2446, S2448 and S2481. Recent reports have shown that p70S6 kinase is a major effector of mTOR phosphorylation at Ser-2448 in response to both mitogen- and nutrient-derived stimuli^52^. Whereas total placental expression of mTOR was unaffected, phosphorylation of mTOR at Ser-2448 was inhibited by folate deficiency in mice (Fig. 3a,b). Next, we examined the phosphorylation of p70S6K1 and S6 downstream kinases regulated by the mTORC1 complex. Maternal folate deficiency significantly decreased the phosphorylation of S6K (Thr-389) and S6 (Ser-235/236) but did not affect the total expression of any of these kinases (Fig. 3a,b).

In most instances, translation is regulated at the initiation phase, when a ribosome is recruited to the 5′ end of an mRNA. The eIF4E-binding proteins (4E-BPs) inhibit translation initiation by binding to the translation factor eIF4E, and preventing recruitment of the translation machinery to mRNA. However, phosphorylation of the binding protein decreases its affinity for eIF4E, thereby activating protein translation. In mammals, the 4EBP family consists of 3 proteins, 4EBP1, 4EBP2, and 4EBP3 and the best characterized 4EBP is 4EBP1. There are six phosphorylation sites have been identified in 4EBP1 (Thr 37, Thr 46, Ser 65, Thr 70, Ser 83, and Ser 112)^53^ and we studied four of them. Folate deficiency in mice significantly inhibited the phosphorylation of placental 4E-BP1 at Thr-37 and 46 as compared to control. Similarly, phosphorylation of 4E-BP1 at Thr-70 was decreased in placenta of folate deficient mice as compared to control. However, there was no significant differences between the control and folate deficient group placenta, when analyzing 4E-BP1 phosphorylation at Ser-65. Compared to control, total expression of 4E-BP1 was higher in placentas of mice fed the folate deficient diet. Thus, both the increased total expression and decreased phosphorylation of 4E-BP is expected to inhibit protein translation in placentas of folate deficient mice.

### Maternal folate deficiency inhibits placental mTORC2 signaling

**Figure 4:**
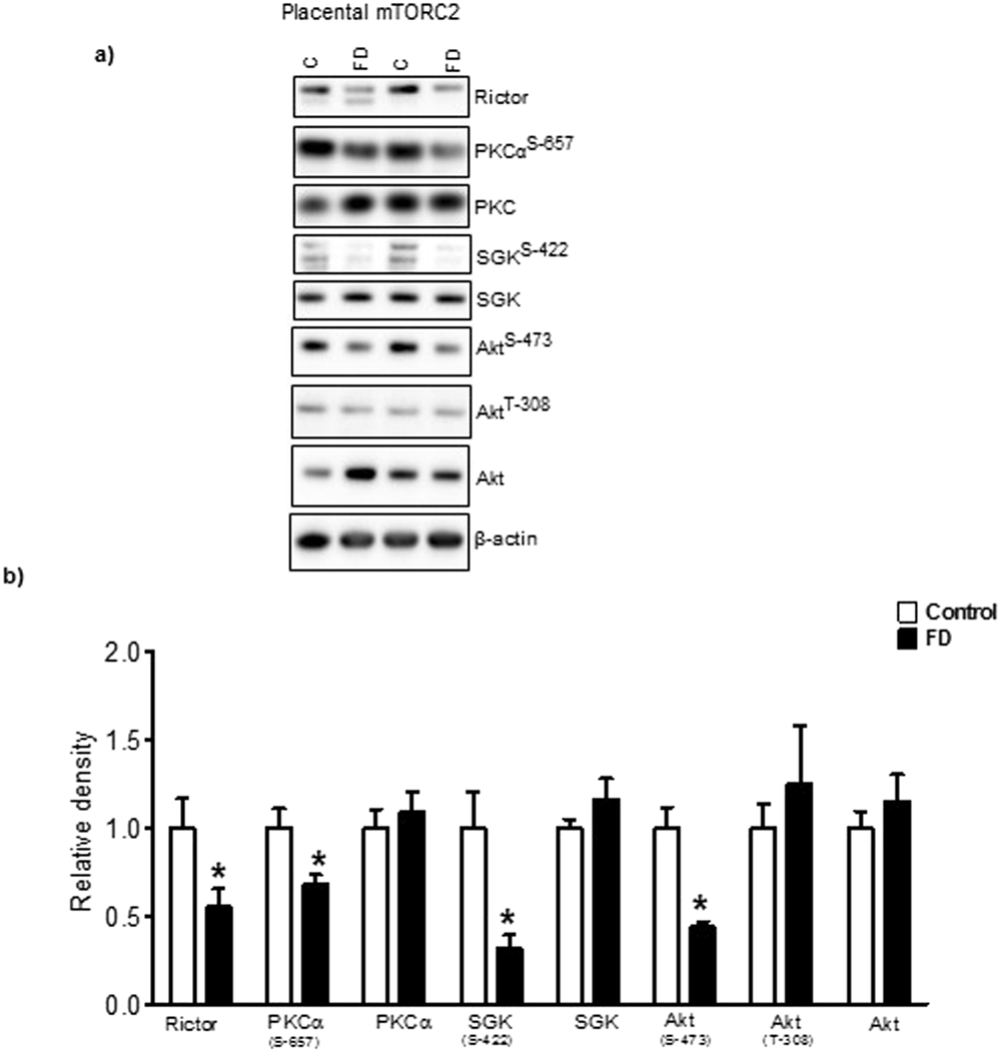
Maternal folate deficiency in mice inhibits placental mTORC2 signaling. (**a**) Placental mTORC2 signaling was inhibited in response to maternal folate deficiency. Histogram (**b**) summarizes the immunoblot data of placental mTORC2. Values are given as mean + SEM; *P < 0.05 vs. control; unpaired Student’s t-test; n = 6–7. Full-length gels and blots are included in the Supplementary Information File.

Rapamycin-insensitive companion of mammalian target of rapamycin (rictor) is a cytosolic protein that was originally recognized as a specific component of mTORC2. An increasing body of evidence however shows that rictor may also function independently of mTORC2 through association with other proteins and complexes^54, 55^. We found that the protein expression of rictor was significantly decreased in placentas of folate deficient dams (Fig. 4a,b). Protein kinase C (PKC) is one of the most extensively studied kinase family and has been implicated in cell proliferation and differentiation^56^. Recent study demonstrates that mTORC2 is involved in post‐translational processing of PKC by facilitating phosphorylation and revealed a novel function of mTORC2 in cellular regulation^57^. The phosphorylation of placental PKCα at Ser-657 was decreased in folate deficiency. There was no significant difference in the total PKCα expression level between control and FD group (Fig. 4a,b).

SGK is a downstream target of mTORC2^55^, serving as a critical node in a highly conserved signaling pathway that integrates multiple inputs from the environment^58^. Activation of SGK1 is triggered by phosphorylation of a threonine residue within the T-loop of the kinase domain and a serine residue lying within the C-terminal hydrophobic motif (Ser422 in SGK1)^58^. Phosphorylation of SGK1 at Ser-422 was significantly decreased in the placenta of folate deficient mice as compared to control. However, total SGK1 expression was comparable between control and folate deficient group mice (Fig. 4a,b).

The activation of Akt involves the phosphorylation of two residues. Threonine 308 (Thr308) in the activation loop of Akt is phosphorylated by PDK1, and therefore represents a functional readout of the insulin/IGF-I growth factor signaling pathway. In contrast, serine 473 (Ser473) in the hydrophobic motif is phosphorylated by mTORC2^59^. The phosphorylation of placental Akt at Ser-473 was markedly decreased in folate deficient mice. Importantly, folate deficiency did not influence Akt phosphorylation at Thr-308 (Fig. 4a,b), suggesting that the impact of low folate on cellular signaling is specific. There was no significant difference in the total Akt expression level between folate deficient and control placentas (Fig. 4a,b).

### Maternal folate deficiency decreases placental amino acid transport activity in mice

We have previously demonstrated that mTOR signaling regulates placental amino acid transport and that mTOR inhibition decreases amino acid transport by preventing the trafficking of specific transporter isoforms to the plasma membrane^27^. To examine the effect of maternal folate deficiency on placental amino acid transport we isolated trophoblast plasma membranes (TPM)^44, 45^ from folate-deficient and control pregnancies. Importantly, the activity of System A and L amino acid transporters was significantly decreased in TPM isolated from folate deficient dams (Supplementary Figure 2a,b). To explore if the marked reduction in TPM amino acid transport activity was associated with a decrease in the transporter abundance, we determined the isoform protein expression by Western blot in isolated TPM. The TPM protein expression of SLC38A2 (Sodium-coupled Neutral Amino acid Transporter 2, SNAT2, an isoform of the System A amino acid transporter) and SLC7A5 (Large neutral Amino acid Transporter, LAT1, a System L amino acid transporter isoform), but not SLC38A1 (SNAT1) or SLC7A8 (LAT2), was markedly decreased in response to maternal folate deficiency (Supplementary Figure 2c,d).

### Folate deficiency in pregnant mice decreases the interaction between mTOR and LAMP2 in the placenta

**Figure 5:**
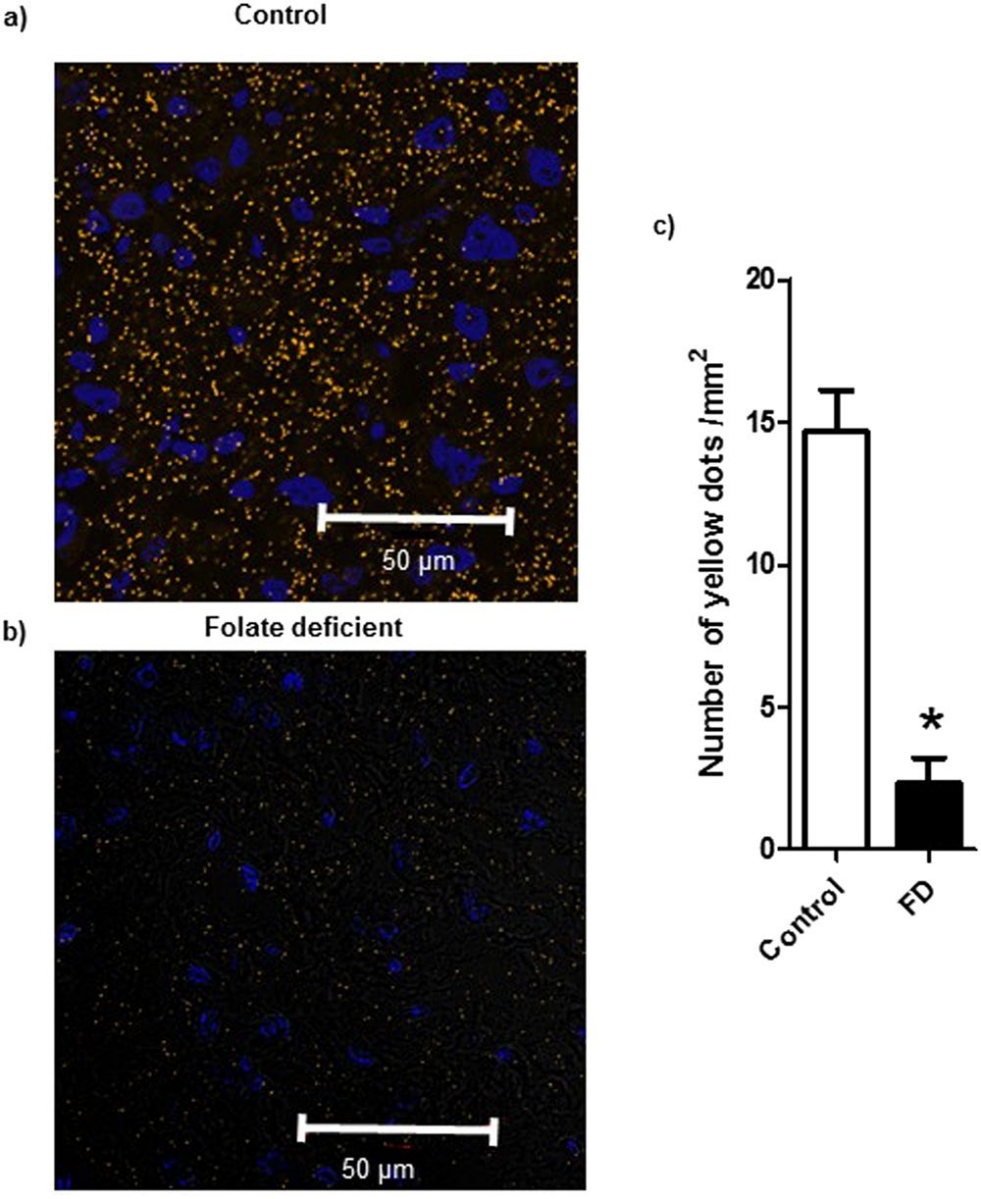
Folate deficiency in pregnant mice decreases the interaction between mTOR and LAMP2 in the placenta. (**a**,**b**) Proximity ligation assay and confocal microscopy was used to determine interactions between mTOR and LAMP2, a lysosomal marker, in the placenta of control and folate deficient pregnant mice. In control placentas (top) mTOR and LAMP2 were in close proximity of each other as evidenced by a large number of yellow dots, suggesting localization of mTOR at the surface of the lysosomes. In contrast, folate deficiency markedly decreased the interaction between mTOR and LAMP2, consistent with localization of mTOR away from the lysosomes. Blue = nuclei; Scale bar-50µm. (**c**) Histogram summarizes the proximity ligation assay data of placental mTOR-LAMP2 interactions. In each section, at least ten randomly selected microscopic fields were used to calculate the number of mTOR-LAMP2 interaction positive sites (yellow dots) per mm^2^ and data were averaged to represent a single placenta. Values are given as mean + SEM; *P < 0.05 vs. control; unpaired Student’s t-test; n = 3 placenta/each group.

Activation of the mTORC1 pathway by amino acids has been shown to promote the translocation of mTORC1 from an unidentified vesicular compartment to a membrane-bound compartment containing Rab7, a marker of both late endosomes and lysosomes^60^. More recent data show that the precise location of mTORC1 is at the lysosomal surface, characterized by LAMP2-positive staining^61^. To test the possibility that folate also regulate the mTOR subcellular localization, we used confocal microscopy and proximity ligation assay to determine the extent of co-localization between mTOR and LAMP2 in placentas of control and folate deficient mice. Whereas mTOR co-localized with LAMP2 in placentas of control mice, the proximity ligation assay signal was markedly attenuated in placentas of folate deficient mice, suggesting a decrease in mTOR/LAMP2 interaction (Fig. 5a,b,c). These data are consistent with the model proposed for mTORC1 amino acid sensing whereby mTORC1, in response to amino acids, is shuttled to the lysosome where it interacts with the nutrisome, resulting in mTORC1 activation^62^.

### Effect of maternal folate deficiency on maternal tissue growth and mTOR signaling

**Figure 6:**
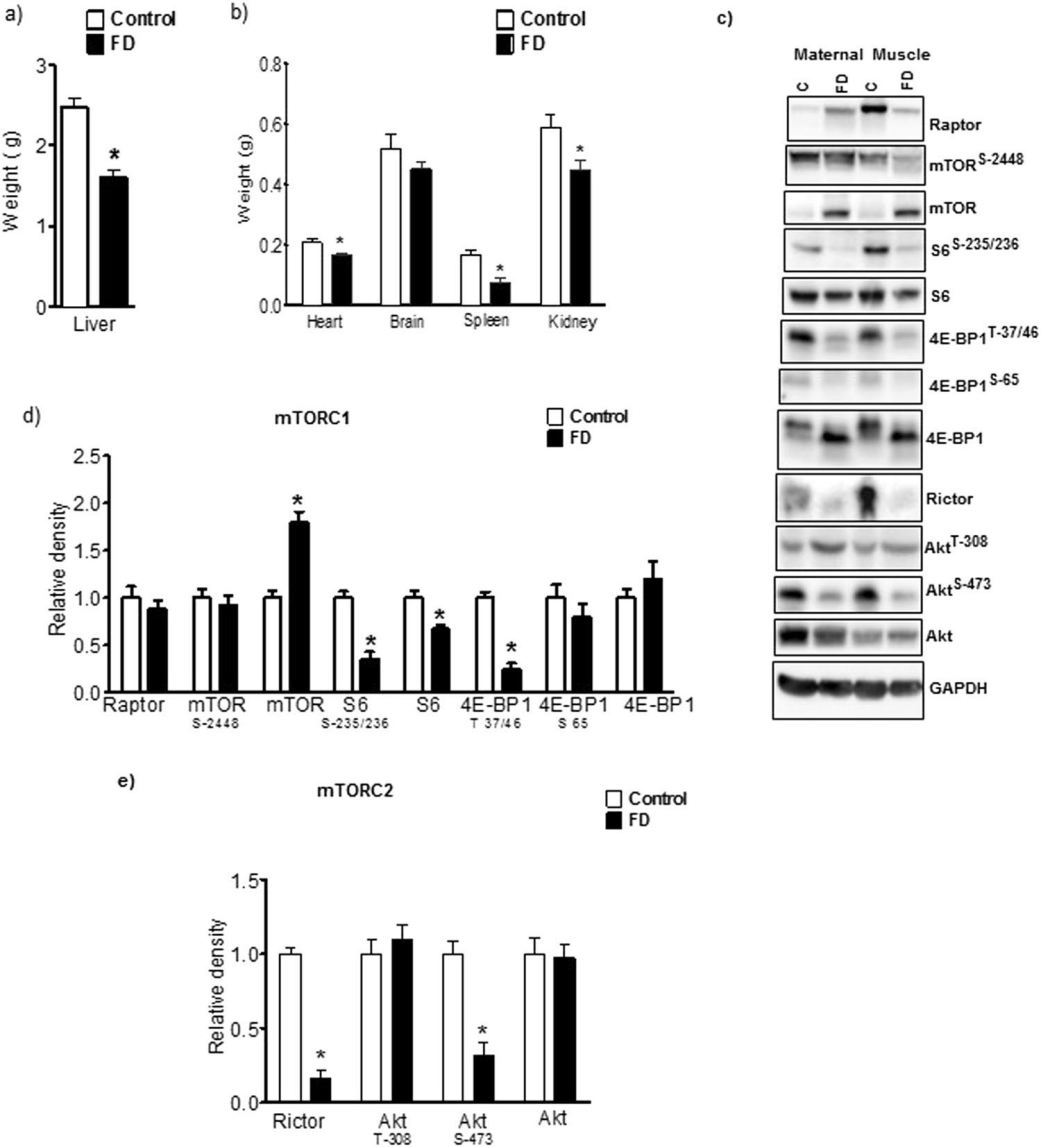
Effect of maternal folate deficiency on maternal tissue growth and mTOR signaling. (**a**) Maternal liver and (**b**) heart, spleen, kidney weights were decreased in folate deficient dams. Values are given as mean + SEM; *P < 0.05 vs. control; unpaired Student’s t-test; n = 6–7. (**c**) mTORC1 (S6-S-235/236, S6, 4E-BP1-T-37/46) and mTORC2 (Rictor, Akt-S-473) downstream signaling in maternal skeletal muscle was inhibited in folate deficient dams. Using Western blot analysis, two distinct bands were observed for total 4E-BP1 in maternal muscle and analyzed both bands together. Histogram (**d**,**e**) summarizes the immunoblot data of maternal skeletal muscle mTORC1and mTORC2 signaling. Values are given as mean + SEM; *P < 0.05 vs. control; unpaired Student’s t-test; n = 6–7. Full-length gels and blots are included in the Supplementary Information File.

Maternal folate deficiency significantly decreased the weights of maternal liver, heart, spleen and kidney (Fig. 6a,b).

#### Maternal muscle

The phosphorylation of ribosomal protein S6 at S-235/236 and 4E-BP1 at T-37/46 were significantly decreased in maternal muscle of FD group as compared to control (Fig. 6c,d,e). However, the phosphorylation of 4E-BP1 at S-65 was unaffected by FD. No significant differences were found in the expression of maternal muscle raptor, total 4E-BP1 expression between control and FD group. In contrast, total S6 expression in maternal muscle was significantly decreased by FD. Furthermore, folate deficiency significantly decreased the expression of rictor and phosphorylation of Akt at S-473. No significant differences were found in the expression of maternal muscle total Akt and phosphorylated Akt-T-308 between control and FD group (Fig. 6c,d,e).

#### Maternal heart

As shown in Supplementary Figure 3(a,b,c), FD significantly decreased the phosphorylation of S6 at S-235/236 and 4E-BP1 at T-37/46 in heart homogenates. No significant differences were found in the expression of maternal heart raptor, total mTOR, S6, 4E-BP1 and phosphorylated mTOR- S-2448 and 4E-BP1-T-70 between control and FD group. In addition, maternal folate deficiency caused a marked decrease in Akt phosphorylation at S-473 in the heart. However, total Akt expression was comparable between FD and control group.

#### Maternal liver

Maternal folate deficiency significantly decreased the phosphorylation of mTOR-S-2448, S6-S-235/236, 4E-BP1-T-37/46 (mTORC1 signaling) and SGK-S-422, Akt-S-473 (mTORC2 signaling) in the liver. In contrast, total mTOR and 4E-BP1 expression were increased in liver homogenates of FD as compared to control. However, total S6 and Akt expression were comparable between FD and control group Supplementary Figure 3(d,e,f).

### Effect of maternal folate deficiency on fetal tissue growth and mTOR signaling

At E18.5, the weights of fetal liver and heart were decreased (Supplementary Figure 4a,b) in response to maternal folate deficiency.

#### Fetal liver

Maternal folate deficiency decreased the expression/phosphorylation of the downstream targets of mTORC1 signaling such as raptor, mTOR-S-2448, S6K-T-389, 4E-BP1-T-70 in the fetal liver (Supplementary Figure 4c). Total mTOR and 4E-BP1 expression was increased in fetal liver of FD group as compared to control. Moreover, maternal folate deficiency significantly decreased the expression/phosphorylation of rictor, SGK-S-422, Akt-S-473 in fetal liver. However, fetal liver S6-S-235/236, total S6K, S6 (mTORC1 signaling) and total SGK, Akt (mTORC2 signaling) expression was comparable between control and FD mice.

#### Fetal heart

FD caused a marked decrease in phosphorylation of mTORC1 downstream targets such as S6-S-235/236, 4E-BP1- T-70, 37/46, 4E-BP1-S-65 and mTORC2 functional downstream targets such as PKC-α-S-657, SGK-S-422, Akt-S-473 in fetal heart (Supplementary Figure 4d). Total 4E-BP1 expression was significantly increased in the heart homogenates of FD group as compared to control. However, fetal heart mTOR-S-2448, total mTOR, S6, rictor, PKC α, SGK, Akt-T-308 expression/phosphorylation was unaffected by FD.

### Fetal kidney

In contrast to fetal liver and heart, mTORC1 (raptor, mTOR-S-2448, mTOR, S6, 4E-BP1 T-70, 37/46, and total 4E-BP1) and mTORC2 (Akt-S-473) signaling were comparable in fetal kidney homogenates between FD and control group mice (Supplementary Figure 4e).

### Maternal serum folate is associated with fetal weight and placental mTORC1 signaling in the baboon

**Figure 7:**
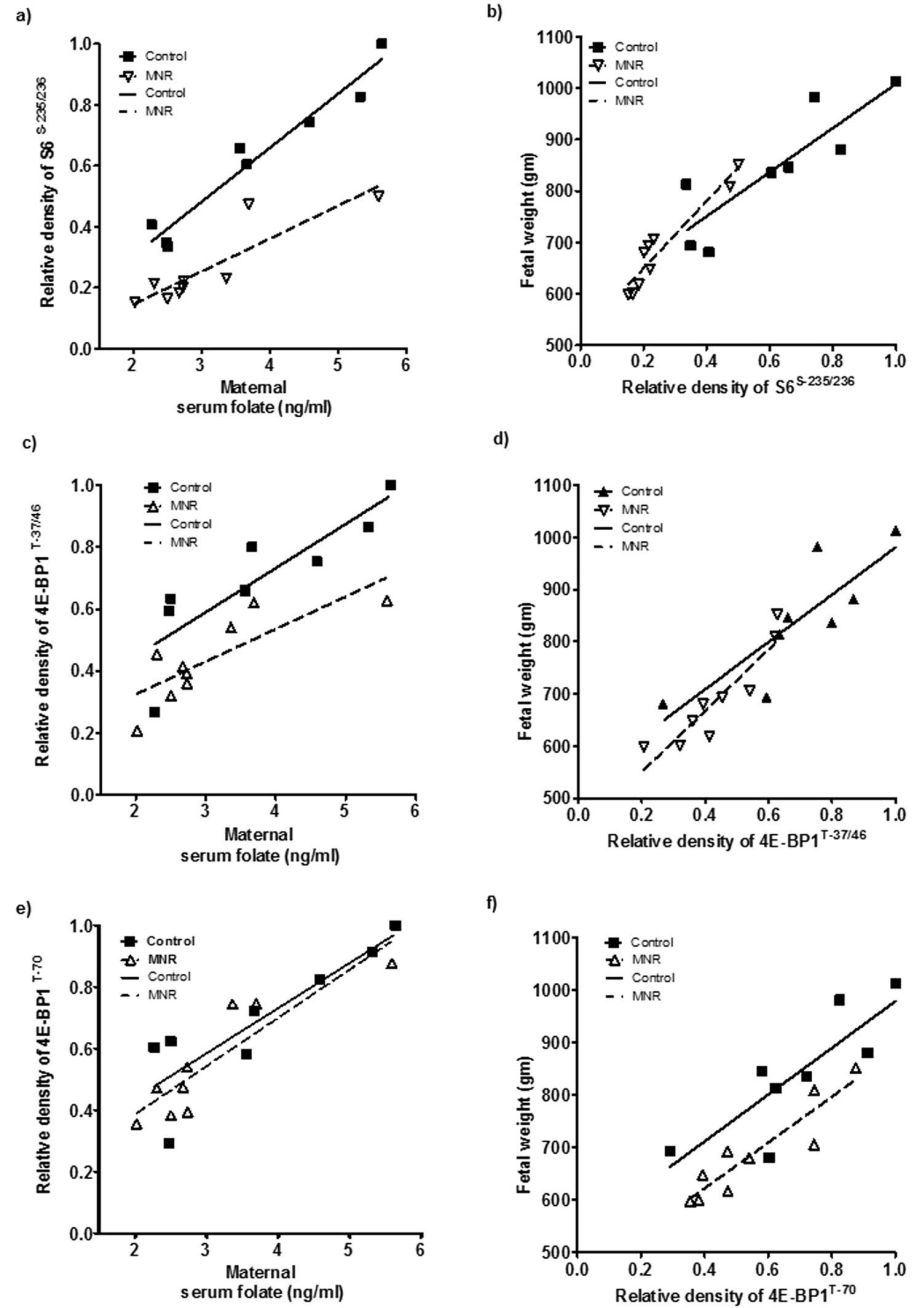
(**a**,**c**,**e**) Correlation between maternal serum folate and placental mTORC1 functional readouts in baboon at gestational day 165. (**a**) Relationship between maternal serum folate and placental S6 (Serine-235/236); Control, r = 0.97, p = 0.0001; MNR, r = 0.88, p = 0.001. (**c**) Relationship between maternal serum folate and placental 4E-BP1 (Threonine-37/46); Control, r = 0.86, p = 0.006; MNR, r = 0.81, p = 0.008. (**c**) Relationship between maternal serum folate and placental 4E-BP1 (Threonine-70); Control, r = 0.87p = 0.005; MNR, r = 0.89, p = 0.001. (**b**,**d**,**f**) Correlation between placental mTORC1 functional readouts and fetal weight in baboon at gestational day 165. (**b**) Relationship between placental S6 (Serine-235/236) and fetal weight; Control, r = 0.86, p = 0.005; MNR, r = 0.96, p = 0.0001. (**d**) Relationship between placental 4E-BP1 (Threonine-37/46) and fetal weight; Control, r = 0.83, p = 0.01; MNR, r = 0.91, p = 0.0006. (**e**) Relationship between placental 4E-BP1 (Threonine-70) and fetal weight; Control, r = 0.83, p = 0.01; MNR, r = 0.92, p = 0.0005. Control, n = 8; MNR, n = 9; r = Pearson’s correlation coefficient.

Next we examined the relationship between maternal serum folate and fetal weight and placental mTORC1 and mTORC2 signaling in baboon pregnancy. Maternal nutrient restriction in the baboon significantly reduced fetal weights (Supplementary Figure 5a). Furthermore, maternal serum folate levels were positively correlated with fetal weights in both the control and MNR group (Supplementary Figure 5b). However, maternal serum folate levels were not significantly different between the control and MNR group (Supplementary Figure 5c). Placental mTORC1 and mTORC2 signaling in the same placentas has been reported elsewhere^34^. Placental mTORC1 signaling functional readouts phosphorylated S6 (Serine-235/236), 4E-BP1 (Thr-37/46) and S6 kinase (Thr-389) were positively correlated with maternal serum folate and fetal weights (Fig. 7a–f).

### Placental microvillus membrane amino acid transport is associated with maternal serum folate and fetal weight in the baboon

Syncytiotrophoblast microvillus plasma membrane SNAT2 and LAT1 expression were positively correlated with maternal serum folate and fetal weights. (Supplementary Figure 6a–d). Similarly, microvillus membrane system A and system L activity were positively correlated with maternal serum folate and fetal weights. (Supplementary Figure 7a–d).

### Maternal serum folate is associated with placental microvillus membrane system L activity in human pregnancy

To explore the clinical relevance of our findings, we examined the relationship between maternal serum folate and placental microvillus membrane system L activity in term placentas from a cohort of healthy women (n = 13) undergoing cesarean section at term. Even in this small cohort, maternal serum folate was positively correlated with birth weight (Supplementary Figure 8a). Furthermore, we observed a significant positive correlation between maternal serum folate levels and placental MVM system L activity (Supplementary Figure 8b). In addition, MVM system L activity positively correlated with birth weight (Supplementary Figure 8c). Phosphorylation of S6 (Serine-235/236), a functional readout of mTORC1 activity, was positively correlated with maternal serum folate (r = 0.7290, p = 0.0047, n = 13). Similarly, phosphorylation of Akt (Serine-473), representing mTORC2 activity, was positively correlated with maternal serum folate (r = 0.7497, p = 0.0032, n = 13).

## Discussion

We show that mTOR functions as a folate sensor *in vivo* in mice extending our recent report that folate is a positive regulator of mTORC1 and mTORC2 signaling in cultured primary human trophoblast cells^43^. Collectively these studies identify a novel mechanism by which folate regulates cell growth and function, which may have broad biological implications. This is also the first study exploring changes in placental signaling and nutrient transporter function and fetal growth in response to maternal folate deficiency in mice. Our findings suggest that placental mTOR folate sensing constitutes a mechanistic link between folate availability and fetal growth, a model supported by the observation that maternal serum folate levels, placental mTOR signaling, nutrient transport and fetal growth are strongly correlated in baboon and human pregnancy.

Although the literature is not entirely consistent^1^, several studies demonstrate a strong positive relationship between maternal red blood cell folate and birth weight^1, 2, 9, 50^. Furthermore, a meta analysis of 8 randomized controlled trials investigating the effect of folate supplementation on fetal growth clearly shows a dose-response relationship between folate intake and birth weight^2^. However, the mechanisms linking folate availability to fetal growth are poorly understood. Fetal growth is largely determined by nutrient availability and trophoblast mTOR signaling is a positive regulator of placental amino acid^26, 27, 63^ and folate transport^64^ and mitochondrial function^65^. mTOR signaling is influenced by an array of upstream regulators, including growth factor signaling, amino acid availability, cellular energy and oxygen levels^40, 41, 66–68^. We recently reported that both mTORC1 and mTORC2 signaling pathways in primary human trophoblast cells are influenced by folate availability with folate deficiency causing a profound inhibition of mTOR signaling activity^43^. The present study provides compelling evidence that mTOR functions as a folate sensor *in vivo* and identifies down-regulation of placental nutrient transporters following inhibition of mTOR signaling as a possible underlying mechanism linking low maternal folate levels to fetal growth restriction. First, maternal folate deficiency in mice resulted in inhibition of placental mTORC1 and mTORC2 signaling pathways, decreased expression of key amino acid transporter isoforms, and lowered placental *in vitro* system A and L amino acid transport activity. Second, maternal folate levels in baboons positively correlated with functional read outs of placental mTORC1 signaling and fetal weights. Third, maternal serum folate correlated positively with birth weight, placental mTORC1 and mTORC2 signaling and placental MVM system L amino acid transport activity in small cohort of pregnant women, suggesting that our finding have clinical relevance. Because trophoblast growth and function plays a critical role in determining fetal growth^69–71^, folate sensing by trophoblast mTOR may represent a mechanism by which maternal folate status modulates fetal growth and development.

It is possible that the effect of maternal folate deficiency on placental function is mediated by indirect effects rather by placental mTOR folate sensing. For example, it cannot be excluded that folate deficiency resulted in changes in maternal circulating levels of metabolic hormones, adipokines and nutrient levels, which are well established to regulate placental function^72^. However, the striking similarity in trophoblast responses to folate deficiency *in vivo* and *in vitro* ^43^ suggest that this is unlikely. Specifically, the signature of mTORC1 and mTORC2 inhibition, including down-regulation of rictor expression and markedly decreased Ser-473 Akt phosphorylation, leaving Ser-308 Akt phosphorylation unaffected was remarkably similar the mouse placenta *in vivo* (present study) and in primary trophoblast cells *in vitro* ^43^. Furthermore, given that we previously have reported that mTORC1 and 2 regulates the plasma membrane trafficking of specific amino acid transporter isoforms (SNAT 2 and LAT1)^27^, the finding that folate deficiency *in vivo* in mice specifically decreased the protein expression of these two isoforms in the trophoblast plasma membrane, suggest that that the down regulation of placental amino acid transport in folate deficient dams are a result of mTORC1 and 2 inhibition.

Folate deficiency resulted in a decreased phosphorylation of specific functional readouts of mTORC1 and mTORC2 signaling in maternal muscle, liver, heart tissues and fetal liver, heart, consistent with the possibility that mTOR is highly sensitive to folate availability in cells other than trophoblasts and that mTOR folate sensing has broad biological significance. For example, antifolates, which are drugs that inhibit the cellular actions of folate by blocking the function of the enzyme dihydrofolate reductase, have been used in cancer treatment for a long time. The presumed action of antifolates is to limit the availability of nucleotides for DNA synthesis in rapidly proliferation cancer cells. In addition, emerging evidence suggest that high folate intake also promotes carcinogenesis^73, 74^, however the underlying molecular mechanism remains elusive. Given the importance of mTOR signalling in promoting cancer cell growth and proliferation and our identification of mTOR as a folate sensor, we speculate that mTOR inhibition contributes to the mechanism of action of antifolates and mTOR activation may link folate excess to carcinogenesis.

Recent advances in the field of mTORC1 amino acid sensing have placed this cellular process at the surface of the lysosome^60–62^. Specifically it is believed that amino acids regulate the recruitment of mTORC1 to the lysosomal surface, where mTORC1 is activated. Using confocal microscopy and proximity ligation assay we provided evidence that mTOR is localized at the lysosomal surface in placentas of control animals. However in placentas collected from folate deficient dams, mTOR was no longer associated to the lysosomes, confirming our previous *in vitro data* in primary human trophoblast cells^43^. These preliminary observations suggest that mTOR amino acid and folate sensing share common mechanisms, however additional studies are required to unravel the molecular mechanisms linking folate to mTOR signaling.

In conclusion, we have identified a novel specific molecular link between maternal folate availability and fetal growth, involving regulation of placental mTOR signaling by folate resulting in changes in placental nutrient transport. We propose that placental mTOR folate sensing cells matches placental nutrient transport and fetal growth to maternal folate status. These findings are clinically relevant because we show that maternal serum folate levels are positively correlated to placental mTOR signaling and nutrient transport and fetal weight in women. mTOR folate sensing may have broad biological significance because of the critical role of folate in normal cell function and the wide range of disorders, including cancer, that have been linked to folate availability.

## Electronic supplementary material

Supplementary Table and Figures
