# Supplementary material for: Maternal folate deficiency causes inhibition of mTOR signaling, down-regulation of placental amino acid transporters and fetal growth restriction in mice
Source: Sci Rep. 2017 Jun 21;7:3982. doi: 10.1038/s41598-017-03888-2 (PMC5479823; doi:10.1038/s41598-017-03888-2)
Supplement: Supplementary file 1 — Supplementary Table and Figures [file 41598_2017_3888_MOESM1_ESM.pdf]

Maternal folate deficiency causes inhibition of mTOR signaling, down-regulation of placental amino acid transporters and fetal growth restriction in mice

Fredrick J. Rosario[1], Peter W. Nathanielsz[2,3] ,Theresa L. Powell[1,4] and Thomas Jansson[1]

[1]Division of Reproductive Sciences, Department of Obstetrics and Gynecology, University of Colorado Anschutz Medical Campus, Aurora, CO; [2]Department of Animal Science, University of Wyoming, Laramie, WY; [3]Southwest National Primate Research Center, San Antonio, TX, and [4]Section of Neonatology, Department of Pediatrics, University of Colorado Anschutz Medical Campus, Aurora, CO

Running title: mTOR functions as a folate sensor

Key words: Folic acid, fetal growth, maternal-fetal exchange, pregnancy

Corresponding author: **Fredrick J. Rosario, PhD**

Division of Reproductive Sciences, Department of Obstetrics and Gynecology,

University of Colorado Anschutz Medical Campus

12700 East 19th Avenue, Aurora, Colorado 80045

Telephone: 303-724-8857

Email: fredrick.joseph@ucdenver.edu

Supplementary Figure 1. (a) Trophoblast plasma membrane (TPM) enrichment. The alkaline phosphatase enrichments in TPM vesicles isolated from control and folate deficient animals were not significantly different (n=6 in each group). Values are given as mean + SEM.

Supplementary Figure 2. Maternal folate deficiency in mice down-regulates placenta amino acid transporter activity and expression. (a) System A and **(b)**, System L activity and **(c)** System A (SNAT2) and System L (LAT1) transporter isoform expression were decreased in trophoblast plasma membranes isolated from folate deficient dams. Histogram (**d**) summarizes the immunoblot data of TPM system A and system L transporter isoform. Values are given as mean + SEM; *P < 0.05 vs. control; unpaired Student's t-test; n=6-7.

Supplementary Figure 3. Inhibition of maternal tissue mTORC1 and mTORC2 signaling in response to folate deficiency. Feeding folate deficient diet 6 weeks prior to and during gestation inhibited maternal heart (**a, b, c**) and liver (**d, e, f**) mTORC1 and mTORC2 signaling. **(a, b, c)** mTORC1 (S6-S-235/236, 4E-BP1-T-37/46) and mTORC2 (Akt-S-473) downstream signaling in maternal heart was inhibited in folate deficient dams. (**d, e, f**) mTORC1 (mTOR-S-2448, S6-S-235/236, 4E-BP1-T-37/46) and mTORC2 (Akt-S-473, SGK-S-422) downstream signaling in maternal liver was inhibited in folate deficient dams. Using Western blot analysis, two distinct bands were observed for total 4E-BP1 and phosphorylated 4E-BP1-T-37/46 in maternal liver and both bands were analyzed together. Values are given as mean + SEM; *P < 0.05 vs. control; unpaired Student's t-test; n=6-7.

Supplementary Figure 4. Effect of maternal folate deficiency on fetal tissue weights and mTOR signaling at E18.5. (a) Feeding folate deficient diet prior to and during gestation decreased the (**a**) fetal heart and (**b**) liver weights. Fetal tissue weights were averaged within each litter and

the litter mean was used as the observation for that dam. Thus, n represents the number of litters. (**c-e)** mTORC1 and mTORC2 signaling in **c,** fetal liver and **d,** heart, but not in **e,** kidney, was inhibited in response to maternal folate deficiency. **(c)** Maternal folate deficiency inhibited mTORC1 (Raptor, mTOR-S-2448, S6K-T-389, 4E-BP1-T-70) and mTORC2 (Rictor, SGK-S-422, Akt-S-473) downstream signaling in fetal liver. Using Western blot analysis, two distinct bands were observed for phosphorylated 4E-BP1-T-70, SGK-S-422 and rictor in fetal liver and both bands were analyzed together. (d) Maternal folate deficiency inhibited mTORC1 (S6-S-235/236, 4E-BP1-T- 37/46 & 70, 65) and mTORC2 (PKCα-S-657, SGK-S-422, Akt-S-473) downstream signaling in fetal heart. Using Western blot analysis, two distinct bands were observed for phosphorylated 4E-BP1-T-37/46 and rictor in fetal heart and both bands were analyzed together. Values are given as mean + SEM; *P < 0.05 vs. control; unpaired Student's t-test; n=7/each group.

Supplementary Figure 5. Correlation between maternal serum folate and fetal weight at gestational day 165 in baboon. (**a**) Fetal weight of control and MNR group baboons. Values are given as mean + SEM; *P < 0.05 vs. control; unpaired Student's t-test (**b**) Relationship between maternal serum folate and fetal weight. Control, n=8; MNR, n =9; Control, r= 0.87, p=0.004; MNR, r=0.88, p=0.001; Control, n=8; MNR, n =9; r =Pearson’s correlation coefficient (c) Maternal serum levels of control and MNR group baboons. Values are given as mean + SEM; Control, n=8; MNR, n =9.

Supplementary Figure 6. (a, c) Correlation between maternal serum folate and placental MVM SNAT2 and LAT1 expression in baboon at gestational day 165. (**a**) Relationship between maternal serum folate and placental MVM SNAT2; Control, r= 0.97, p=0.0001; MNR, r=0.74, p=0.02. (c) Relationship between maternal serum folate and placental MVM LAT1;

Control, r= 0.95, p=0.0002; MNR, r=0.96, p=0.0001. (**b, d) Correlation between placental MVM SNAT2 and LAT1 expression and fetal weight in baboon at gestational day 165**. (**b**) Relationship between placental MVM SNAT2 and fetal weight; Control, r= 0.94, p=0.0004; MNR, r=0.80, p=0.009. (d) Relationship between placental MVM LAT1 and fetal weight; Control, r= 0.92, p=0.001; MNR, r=0.95, p=0.0001. Control, n=8; MNR, n =9; r=Pearson's correlation coefficient.

Supplementary Figure 7. (a, c) Correlation between maternal serum folate and placental MVM system A and system L activity in baboon at gestational day 165. (**a**) Relationship between maternal serum folate and placental system A activity; Control, r= 0.94, p=0.001; MNR, r=0.95, p=0.001. (c) Relationship between maternal serum folate and placental system L activity; Control, r= 0.82, p=0.03; MNR, r=0.85, p=0.02. (**b, d) Correlation between placental MVM system A and system L activity and fetal weight in baboon at gestational day 165**. (**b**) Relationship between placental MVM system A and fetal weight; Control, r= 0.86, p=0.01; MNR, r=0.93, p=0.003. (d) Relationship between placental MVM system L and fetal weight; Control, r= 0.84, p=0.02; MNR, r=0.83., p=0.02. Control, n=7; MNR, n =7; r=Pearson's correlation coefficient.

Supplementary Figure 8. (a) Correlation between maternal serum folate and fetal weight in human pregnancy at term. Relationship between maternal serum folate and fetal weight; r= 0.59, p=0.03. (**b) Correlation between maternal serum folate and placental MVM system L activity in human pregnancy at term.** Relationship between maternal serum folate and placental MVM system L activity; r= 0.93, p=0.0001. **(c) Correlation between placental MVM system L activity and fetal weight in human pregnancy at term.** Relationship between placental MVM system L activity and fetal weight. r= 0.51, p=0.04; n =13; r=Pearson's correlation coefficient.

Supplementary Table-1

Dietary composition of Purina monkey diet.

Nutrients[1]

Protein, % 15.6

Arginine, % 0.90
Cystine, % 0.31
Glycine, % 0.67
Histidine, % 0.41
Isoleucine, % 0.63
Leucine, % 1.41
Lysine, % 0.75
Methionine, % 0.42
Phenylalanine, % 0.72
Tyrosine, % 0.46
Threonine, % 0.57
Tryptophan, % 0.16
Valine, % 0.73
Serine, % 0.81
Aspartic Acid, % 1.62
Glutamic Acid, % 3.68
Alanine, % 1.04
Proline, % 1.30
Taurine, % ………….<0.01

Fat (ether extract), % 5.0

Fat (acid hydrolysis), % 6.3

Cholesterol, ppm 80
Linoleic Acid, % 1.46
Linolenic Acid, % 0.09
Arachidonic Acid, % ……<0.01
Omega-3 Fatty Acids, % 0.15
Total Saturated Fatty Acids, % .1.36
Total Monounsaturated Fatty Acids, % 1.72

Fiber (Crude), % 4.2

Neutral Detergent Fiber[2], % . . .15.5
Acid Detergent Fiber[3], % 5.3

Nitrogen-Free Extract (by difference), % 60.0

Starch, % 38.1
Glucose, % 1.01
Fructose, % 1.05
Sucrose, % 2.37
Lactose, % 1.68

Total Digestible Nutrients, % . .78.2

Gross Energy, kcal/gm...........4.08

Physiological Fuel Value[4], kcal/gm 3.47

Metabolizable Energy, kcal/gm....................................3.22

Minerals

Ash, % 5.2

Calcium, % 0.90
Phosphorus, % 0.60
Phosphorus (non-phytate), % . .0.33
Potassium, % 0.75
Magnesium, % 0.18
Sulfur, % 0.24
Sodium, % 0.25
Chloride, % 0.37
Fluorine, ppm…………………19
Iron, ppm 220
Zinc, ppm………………………110
Manganese, ppm…………………97
Copper, ppm………………….21
Cobalt, ppm 0.53
Iodine, ppm……………………1.3
Chromium (added), ppm……..0.01
Selenium, ppm…………………0.37

Vitamins

Carotene, ppm…………………..1.7
Vitamin K, ppm…………………3.2
Thiamin Hydrochloride, ppm…………………………..8.3
Riboflavin, ppm……………….8.6
Niacin, ppm 113
Pantothenic Acid, ppm…………60
Choline Chloride, ppm 1200
<u>Folic Acid, ppm……………..7.9</u>
Pyridoxine, ppm……………….14
Biotin, ppm…………………… 0.1
B_{12}, mcg/kg 73
Vitamin A, IU/gm……………20
Vitamin D_3 (added), IU/gm 6.7
Vitamin E, IU/kg 110
Ascorbic Acid, mg/gm……….0.50

Calories provided by:

Protein, %……………….17.959
Fat (ether extract), %…....12.951
Carbohydrates, %…………69.090

1. Nutrients expressed as percent of ration except where otherwise indicated. Moisture content is assumed to be 10.0% for the purpose of calculations.
2. NDF = approximately cellulose, hemi-cellulose and lignin.
3. ADF = approximately cellulose and lignin.
4. Physiological Fuel Value (kcal/gm) = Sum of decimal fractions of protein, fat and carbohydrate (use Nitrogen Free Extract) x 4,9,4 kcal/gm respectively.

a)

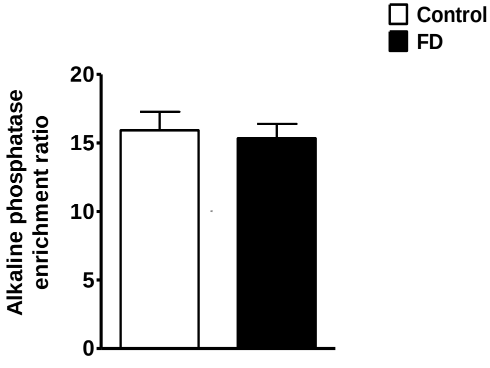

Supplementary Figure -1.

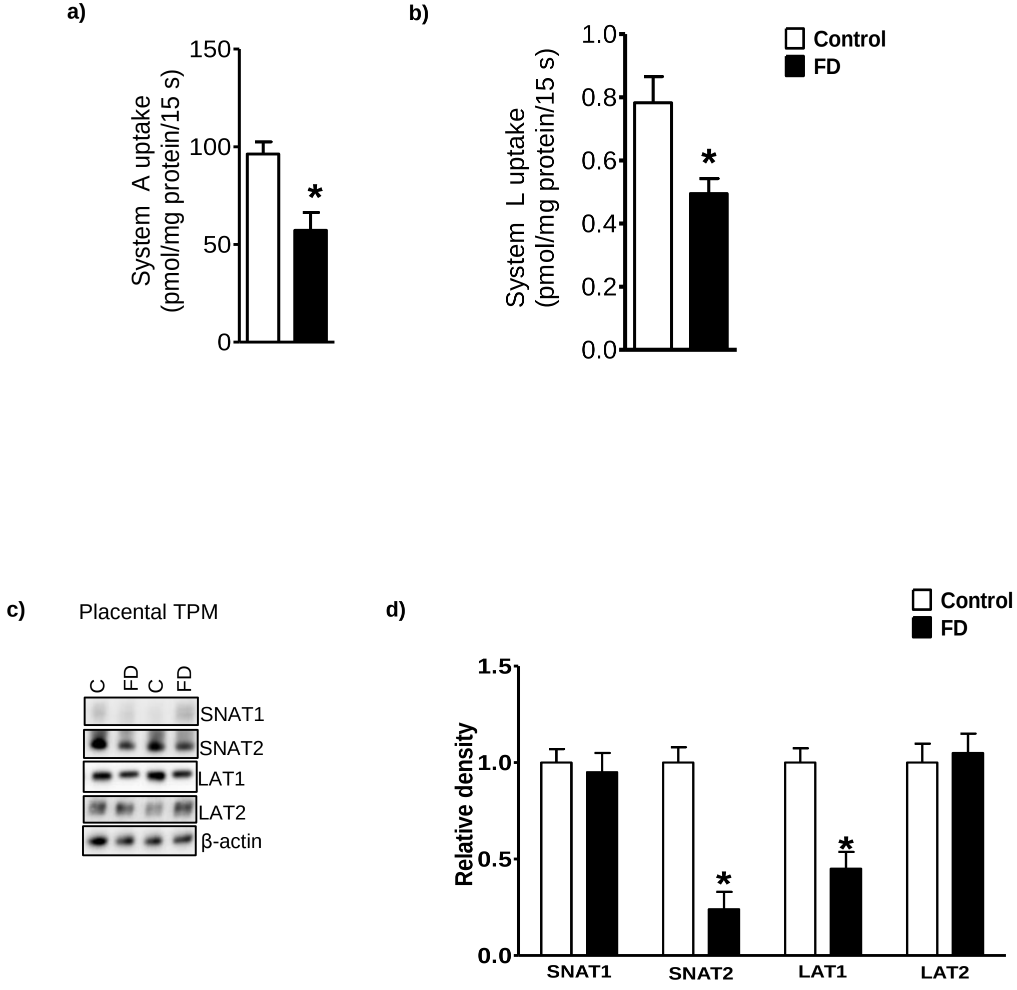

Supplementary Figure -2.

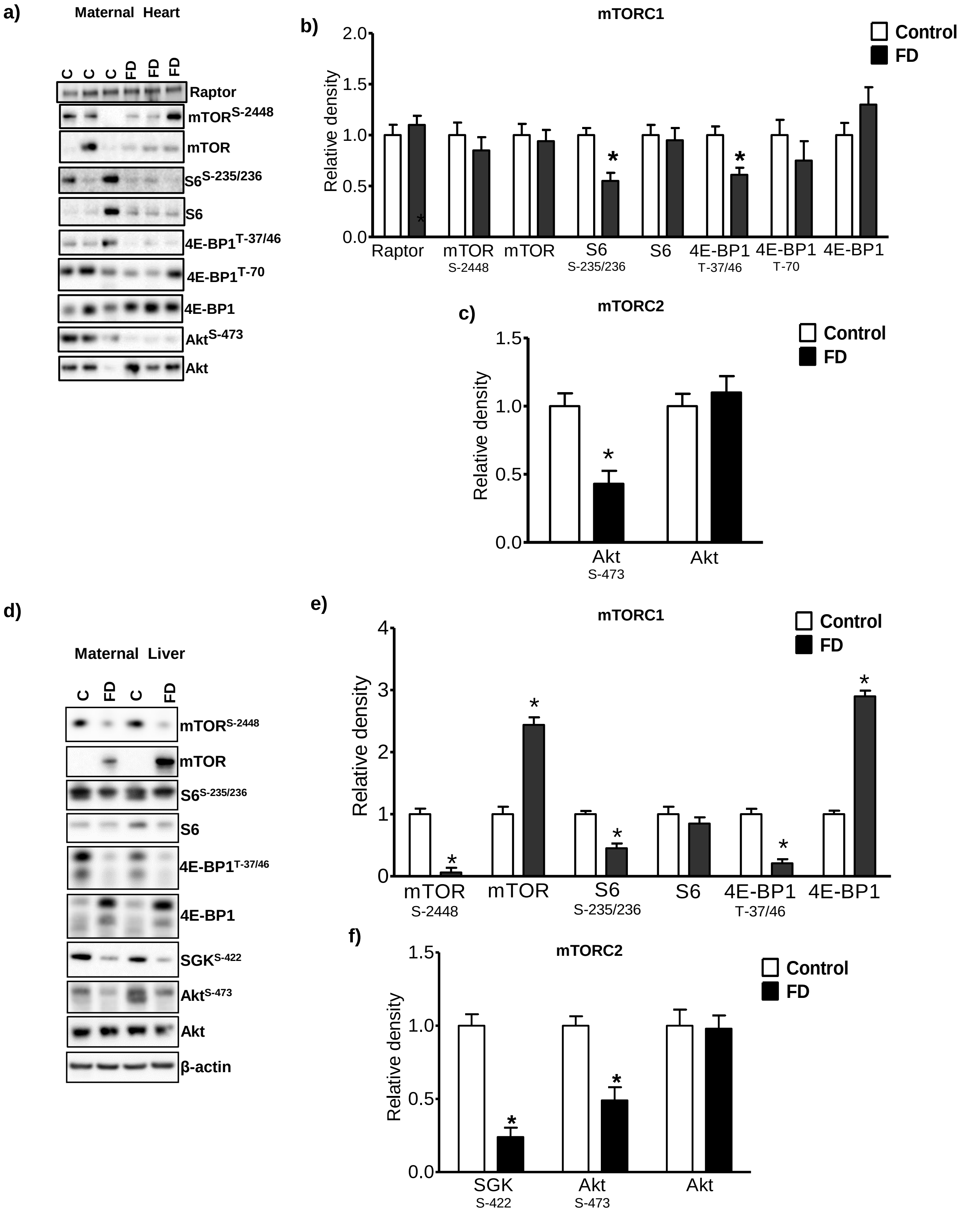

Supplementary Figure -3.

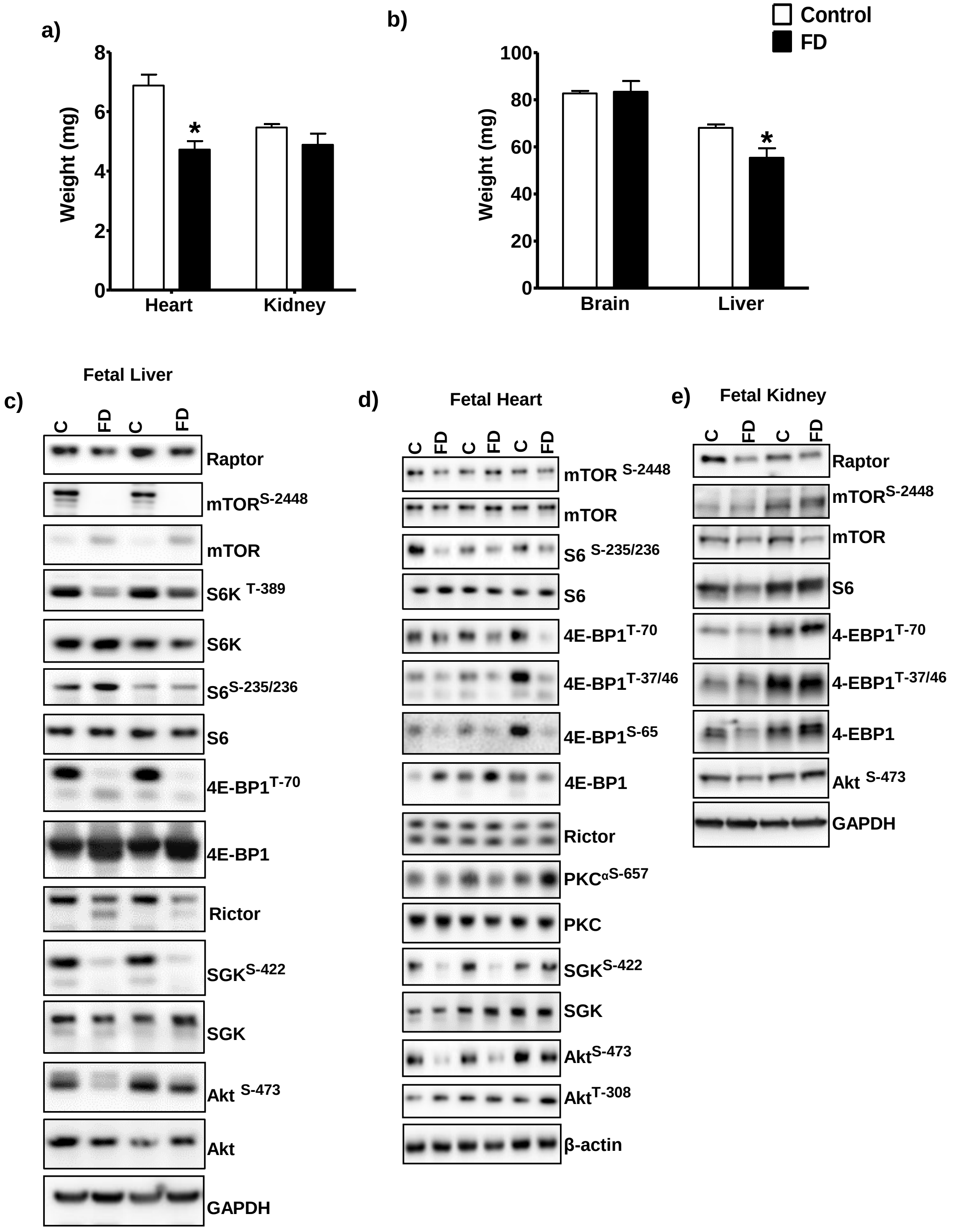

Supplementary Figure -4.

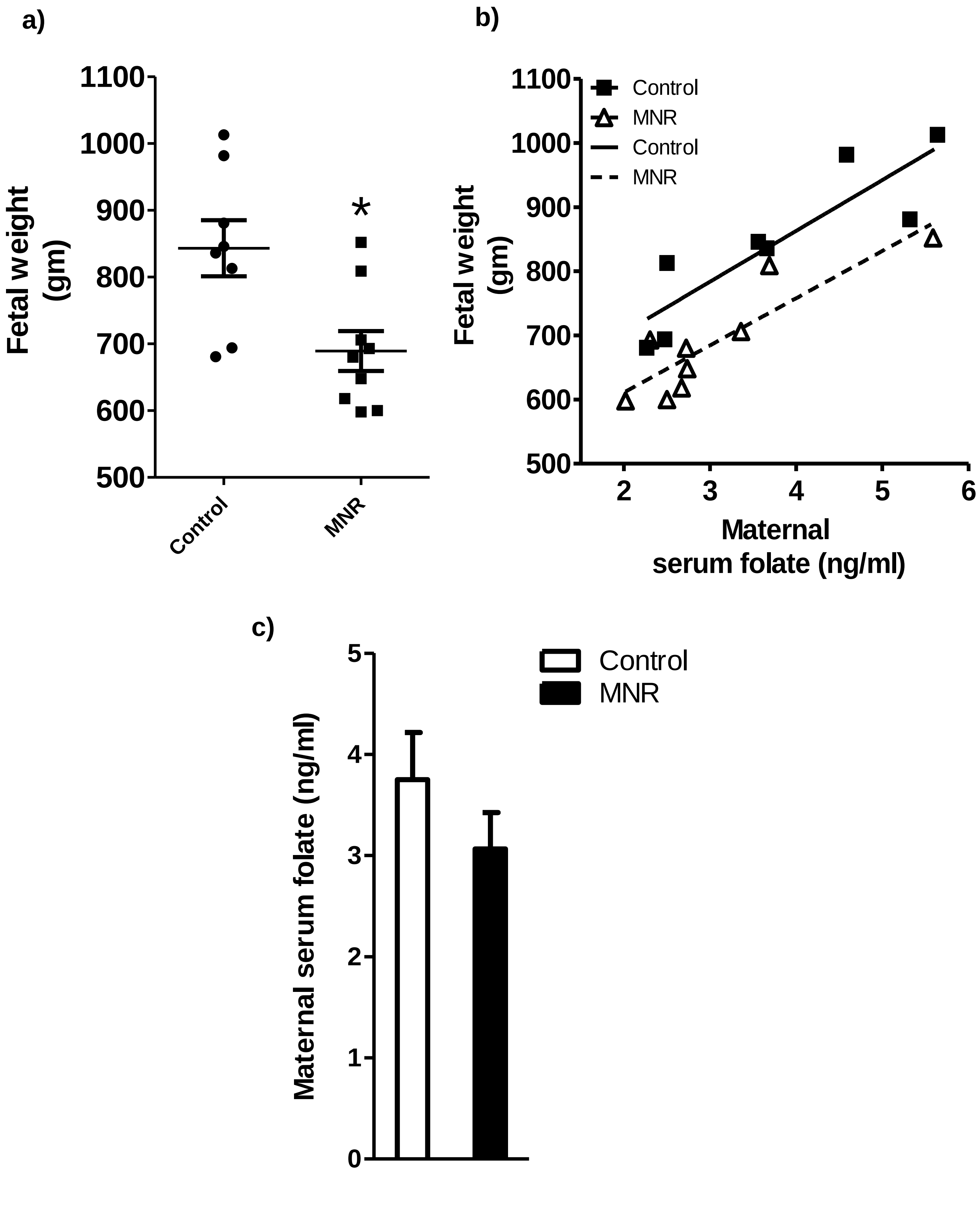

Supplementary Figure -5.

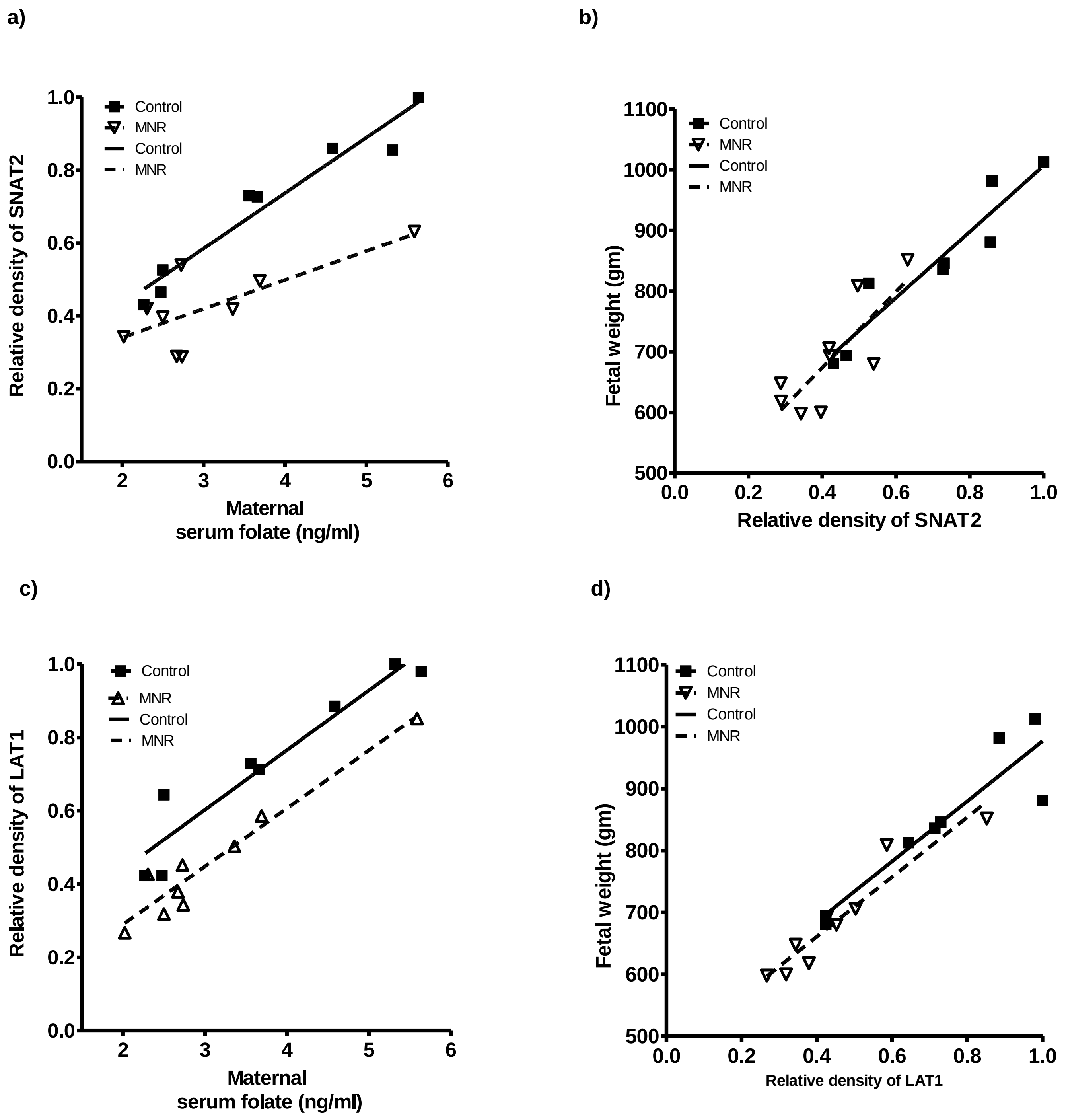

Supplementary Figure 6.

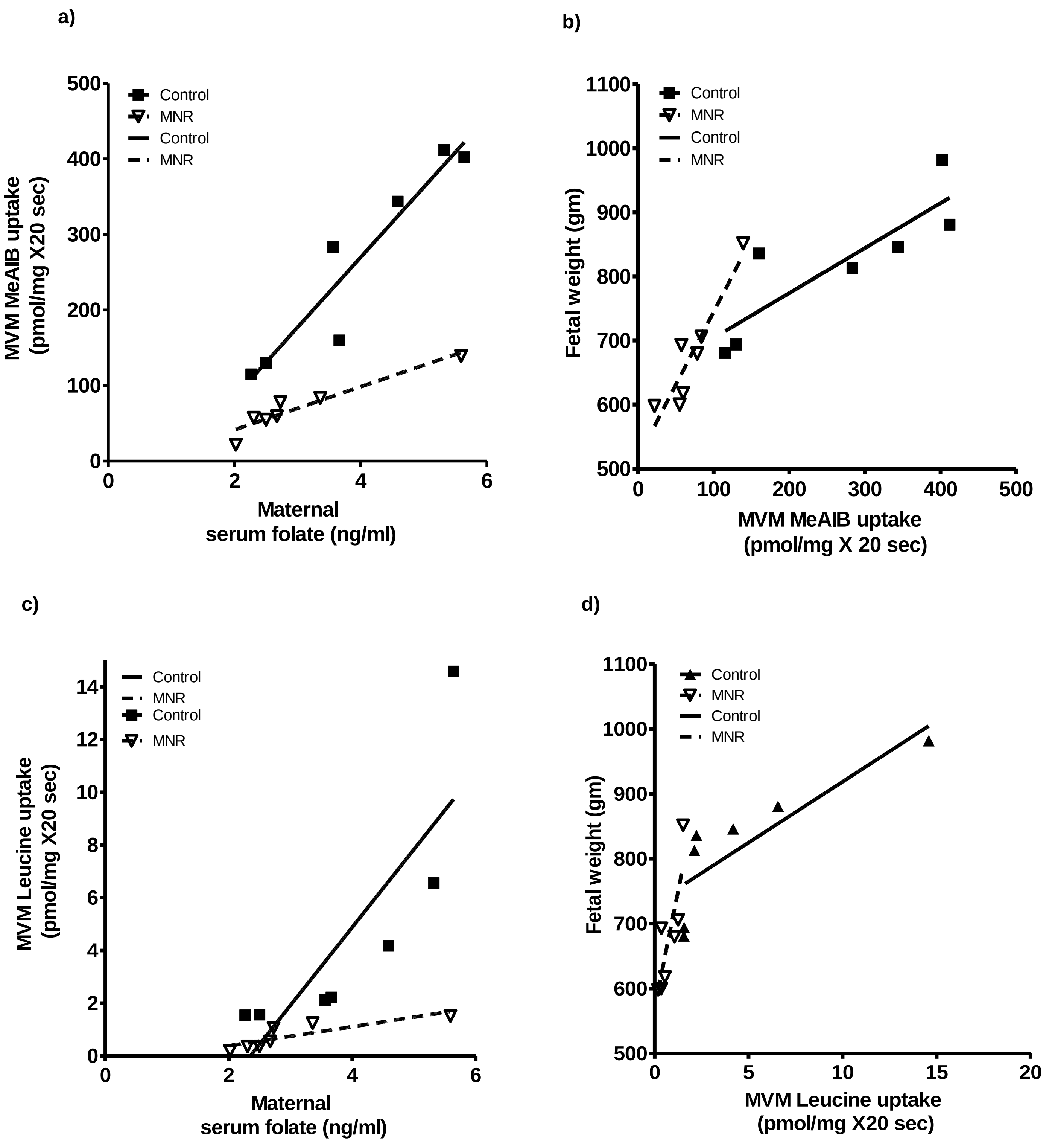

Supplementary Figure 7.

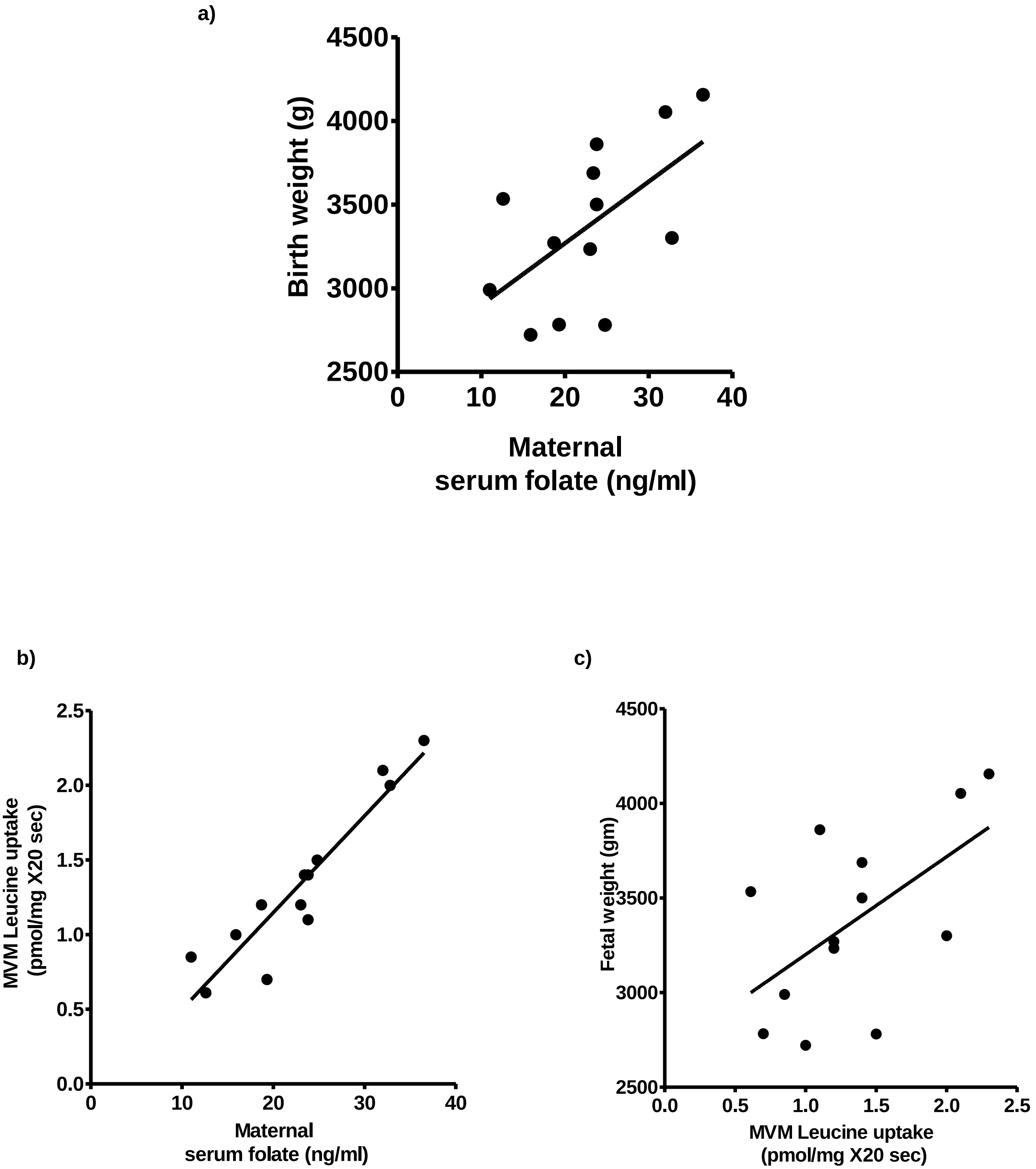

Supplementary Figure 8.

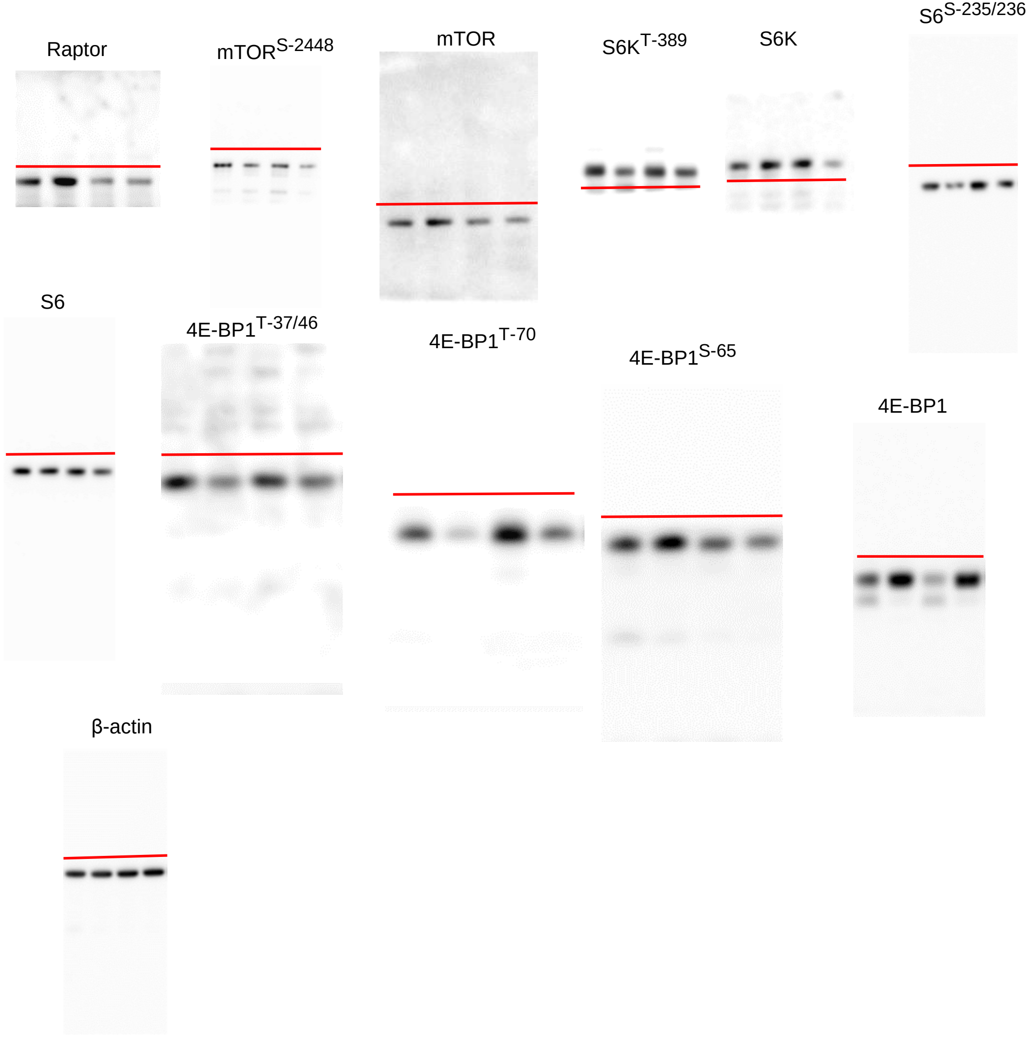

Supplementary Figure 9- Full blots for Figure-3

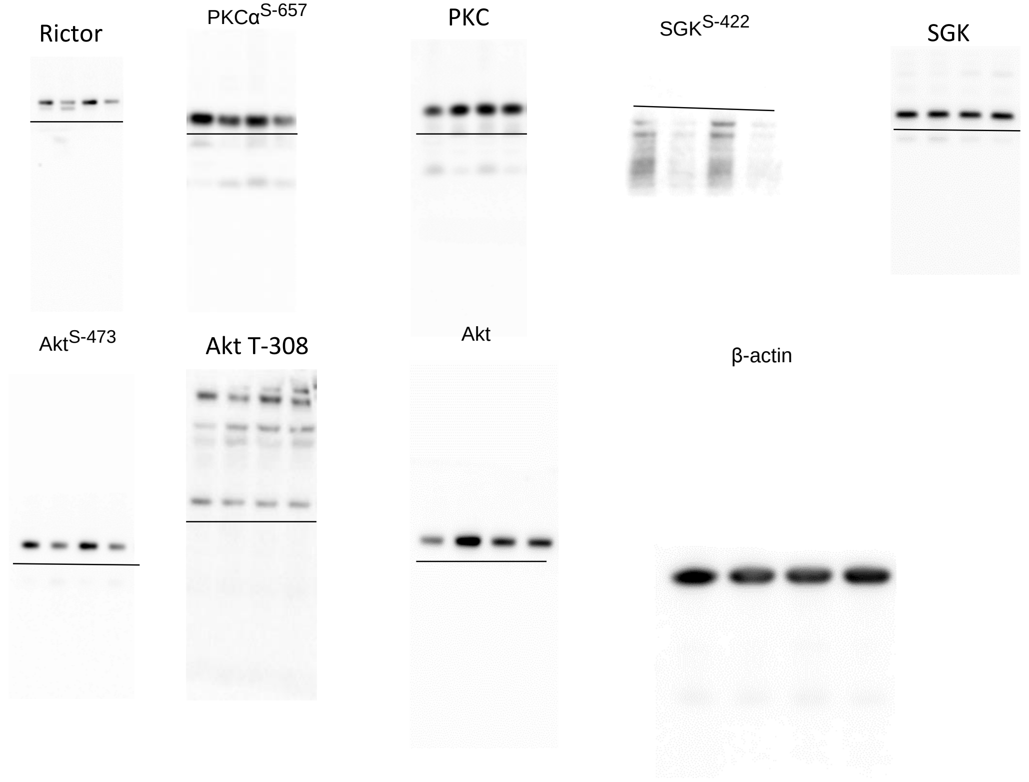

Supplementary Figure 10- Full blots for Figure-4

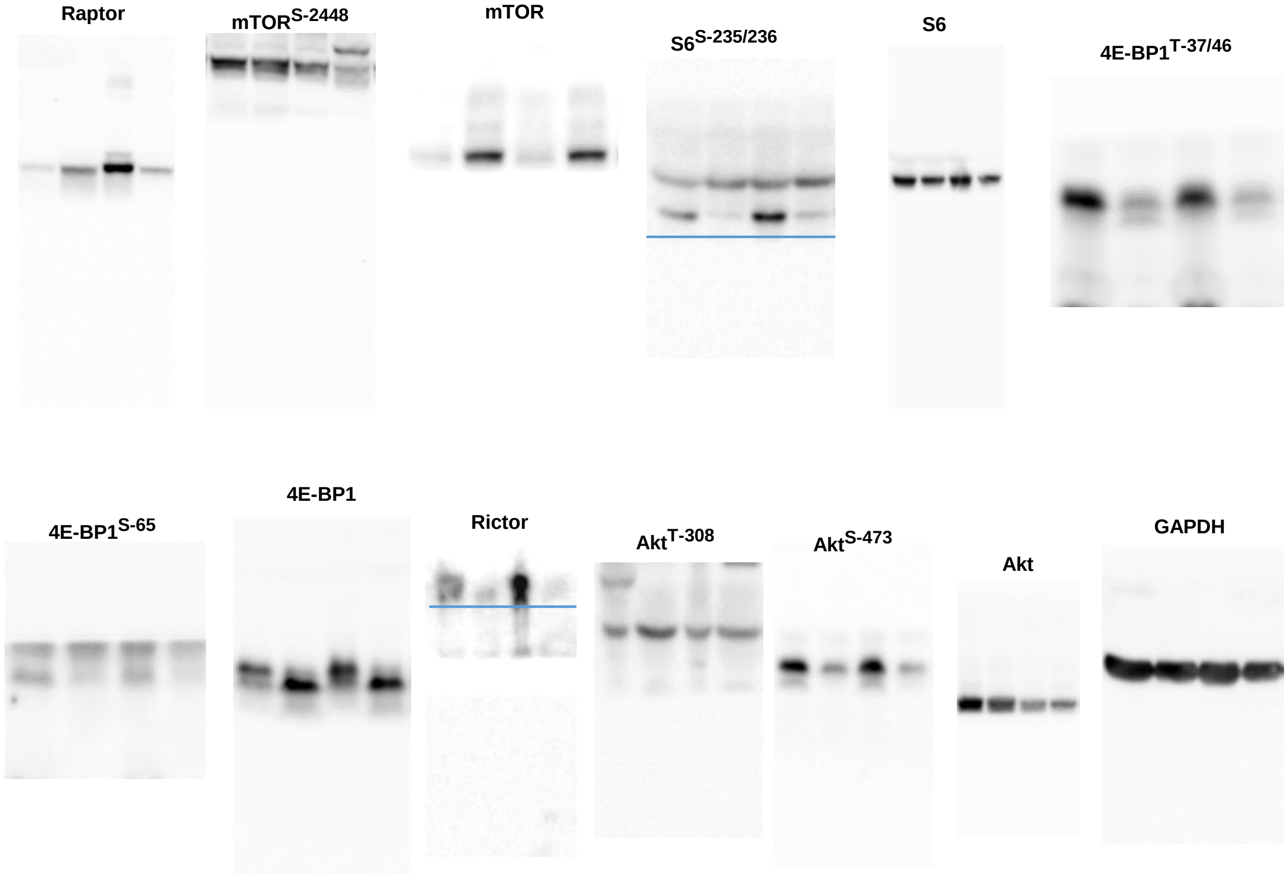

Supplementary Figure 11 Full blots for Figure-6
